# Rosmarinic Acid Sensitizes Ovarian Cancer Cells to Gemcitabine Through Oxidative Stress-Associated Apoptotic and Antiproliferative Responses

**DOI:** 10.3390/ijms27156741

**Published:** 2026-07-28

**Authors:** Coşkun Orhaner, Mehmet Cudi Tuncer, İlhan Özdemir

**Affiliations:** 1 Department of Gynecology and Obstetrics, Soranus IVF Center, 16070 Bursa, Turkey; orhanercoskun@gmail.com; 2 Department of Anatomy, Faculty of Medicine, Dicle University, 21280 Diyarbakır, Turkey; 3 Department of Histology Embryology, Faculty of Medicine, Kahramanmaraş Sütçü İmam University, 46000 Kahramanmaraş, Turkey; ilhanozdemir32@hotmail.com

**Keywords:** rosmarinic acid, OVCAR3, oxidative stress, cell viability, synergism, anticancer

## Abstract

Rosmarinic acid (RA), a naturally occurring polyphenolic compound, has attracted increasing attention because of its potential anticancer activity and capacity to modulate oxidative stress-associated signaling pathways. In the present study, the cytotoxic, apoptotic, and antiproliferative effects of RA, alone or in combination with gemcitabine (Gem), were investigated in OVCAR3 ovarian cancer cells and HaCaT keratinocytes using integrated two-dimensional and three-dimensional (3D) experimental models. Cell viability assays demonstrated dose- and time-dependent growth inhibition following RA and Gem treatment, while combination index (CI) analysis revealed synergistic cytotoxic activity in OVCAR3 cells. Flow cytometric analyses showed that combined treatment markedly increased apoptotic cell populations and altered cell cycle progression through enhanced S-phase and G2/M accumulation. Intracellular reactive oxygen species (ROS) levels were significantly elevated following combination treatment, and N-acetyl-L-cysteine (NAC) pretreatment partially attenuated both ROS accumulation and cytotoxicity, indicating a functional contribution of oxidative stress to the observed antitumor response. RT-qPCR analyses demonstrated increased expression of proapoptotic genes (*BAX*, *CASP3*, and *CASP9*) together with suppression of *BCL2*, *MKI67*, and *CDK4* expression, while immunocytochemical analyses supported enhanced caspase-3 activation at the protein level. In 3D OVCAR3 tumor spheroids, the RA + Gem combination significantly reduced spheroid viability, disrupted spheroid architecture, and increased dead-cell accumulation compared with single-agent treatments. Collectively, these findings suggest that RA may enhance the anticancer activity of Gem in ovarian cancer cells through mechanisms associated with oxidative stress, apoptosis, and proliferation-related signaling pathways under both monolayer and 3D culture conditions.

## 1. Introduction

Ovarian cancer remains one of the leading causes of gynecological cancer-related mortality worldwide because of its late-stage diagnosis, aggressive clinical course, and frequent development of chemoresistance [1,2]. In most patients, the disease is diagnosed only after extensive dissemination to the peritoneal cavity or distant organs, resulting in poor long-term survival despite advances in surgical management and systemic chemotherapy [3]. Although platinum-based chemotherapy combined with taxanes constitutes the current standard treatment approach, recurrence and acquired resistance remain major clinical obstacles that substantially limit therapeutic success. In addition to treatment resistance, cumulative systemic toxicity associated with repeated chemotherapy cycles frequently compromises patient quality of life and restricts long-term treatment efficacy [4]. Therefore, identifying adjunctive therapeutic strategies capable of enhancing anticancer responses while minimizing treatment-associated toxicity has become an important focus of contemporary ovarian cancer research [5].

Gem, a nucleoside analog belonging to the antimetabolite class, exerts its cytotoxic activity primarily through inhibition of DNA synthesis and disruption of cell cycle progression. Following intracellular phosphorylation, Gem interferes with DNA replication and promotes replication stress, ultimately leading to growth arrest and apoptotic cell death [6]. In clinical oncology, Gem is widely used either as monotherapy or in combination regimens for the treatment of several solid malignancies, including pancreatic, lung, bladder, breast, and ovarian cancers [6,7]. In recurrent ovarian cancer, Gem-containing regimens are particularly considered in platinum-resistant or partially platinum-sensitive disease settings [8]. However, hematological toxicity, neuropathy, treatment-associated adverse effects, and the emergence of chemoresistance substantially reduce the long-term therapeutic benefit of Gem-based therapies [9]. These limitations have stimulated growing interest in naturally derived compounds that may enhance chemosensitivity and improve therapeutic responses when combined with conventional anticancer agents.

Natural polyphenolic compounds have attracted considerable attention because of their broad spectrum of biological activities and their potential role in cancer prevention and treatment. Increasing evidence indicates that many naturally occurring polyphenols can modulate oxidative stress, inflammatory signaling, apoptosis, mitochondrial dysfunction, and cell cycle regulation in tumor cells [10,11]. Importantly, several polyphenols have been reported to enhance the efficacy of standard chemotherapeutic agents through additive or synergistic interactions while potentially reducing the required therapeutic dose of cytotoxic drugs. Such multimodal biological activity has positioned natural compounds as promising candidates for combination-based anticancer strategies [12,13].

RA, a hydroxycinnamic acid derivative abundantly present in plants belonging to the Lamiaceae family, including rosemary, sage, basil, and oregano, has emerged as a bioactive polyphenol with diverse pharmacological properties [14]. RA has been extensively investigated because of its antioxidant, anti-inflammatory, antimicrobial, neuroprotective, and anticancer activities [15]. Previous experimental studies have demonstrated that RA may suppress tumor cell proliferation and promote apoptotic responses in multiple cancer models, including breast, endometrial, liver, lung, colorectal, and prostate cancers. Proposed mechanisms associated with RA-mediated anticancer activity include modulation of intracellular ROS levels, activation of mitochondrial apoptotic signaling, regulation of caspase-associated pathways, suppression of proliferation-related signaling, and alteration of cell cycle progression [14,15,16]. In addition, several reports have suggested that RA may influence redox homeostasis in a context-dependent manner, functioning as an antioxidant in non-malignant cells while promoting pro-oxidative stress responses in cancer cells under certain experimental conditions [17].

The OVCAR3 ovarian adenocarcinoma cell line is widely used as an in vitro model for investigating ovarian cancer biology and chemotherapeutic resistance because of its clinically relevant molecular characteristics and resistance-associated phenotype [18]. In the present study, HaCaT keratinocytes were included as a non-malignant epithelial comparator model to evaluate relative differences in treatment-associated cytotoxicity and oxidative stress responses [19]. Although HaCaT cells do not originate from ovarian tissue and therefore cannot fully represent normal ovarian epithelial cells, they were included as a well-established non-malignant epithelial reference model to provide a preliminary assessment of differential treatment-associated responses between malignant and non-malignant cells. Accordingly, the findings obtained using HaCaT cells should be interpreted as an initial evaluation of relative cellular sensitivity rather than definitive evidence of ovarian cancer-specific selectivity [20,21]. While the individual anticancer effects of RA and Gem have been previously investigated, studies evaluating their combined activity in ovarian cancer models remain limited, particularly in the context of ROS-associated cytotoxicity and 3D tumor biology. Moreover, most previous studies examining natural compound–chemotherapy combinations have primarily relied on two-dimensional monolayer systems, which only partially reflect the structural and microenvironmental complexity of solid tumors.

Therefore, the present study aimed to investigate the cytotoxic, apoptotic, and antiproliferative effects of RA and Gem, individually and in combination, using integrated two-dimensional monolayer cultures and 3D OVCAR3 tumor spheroid models. In addition, the potential contribution of oxidative stress-associated mechanisms was evaluated through NAC-based functional rescue experiments together with molecular, immunocytochemical, and bioinformatic analyses. By integrating conventional monolayer assays with 3D tumor modeling, the present study sought to provide a more comprehensive evaluation of the biological effects associated with the RA–Gem combination in ovarian cancer cells.

## 2. Results

### 2.1. Dose- and Time-Dependent Effects of RA and Gem on OVCAR3 and HaCaT Cell Viability

**Figure 1:**
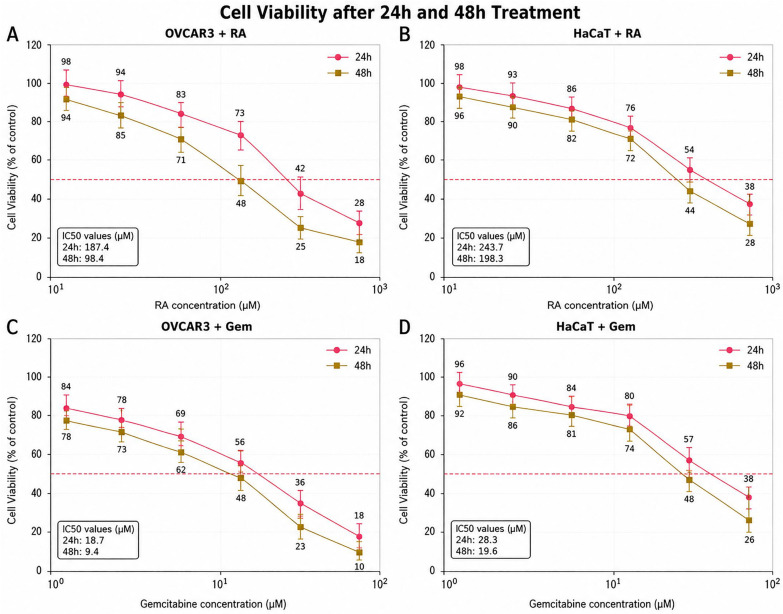
Dose- and time-dependent effects of RA and Gem on cell viability in OVCAR3 and HaCaT cells determined by MTT assay. (**A**) OVCAR3 cells treated with RA. (**B**) HaCaT cells treated with RA. (**C**) OVCAR3 cells treated with Gem. (**D**) HaCaT cells treated with Gem. Cell viability was assessed after 24 h and 48 h treatment exposure and expressed relative to untreated controls. Calculated IC50 values were 187.4 µM (24 h) and 98.4 µM (48 h) for RA-treated OVCAR3 cells, 243.7 µM (24 h) and 198.3 µM (48 h) for RA-treated HaCaT cells, 18.7 µM (24 h) and 9.4 µM (48 h) for Gem-treated OVCAR3 cells, and 28.3 µM (24 h) and 19.6 µM (48 h) for Gem-treated HaCaT cells. Data are presented as mean ± SD (*n* = 3). The red dashed horizontal line indicates the 50% cell viability level used for IC_50_ determination.

The cytotoxic effects of RA and Gem were evaluated in OVCAR3 and HaCaT cells using the MTT assay following 24 h and 48 h treatment periods. Both compounds significantly reduced cell viability in a concentration- and time-dependent manner in both cell lines compared with untreated control groups (*p* < 0.05; Figure 1).

In OVCAR3 cells, RA treatment resulted in half-maximal inhibitory concentration (IC_50_) values of 187.4 µM at 24 h and 98.4 µM at 48 h, whereas the corresponding IC_50_ values in HaCaT cells were 243.7 µM and 198.3 µM, respectively (Figure 1A,B). The greater reduction in viability observed in OVCAR3 cells suggests increased sensitivity of ovarian cancer cells to RA treatment under the tested experimental conditions.

Similarly, Gem treatment significantly decreased cell viability in both cell lines in a dose- and time-dependent manner. In OVCAR3 cells, the IC_50_ values for Gem were calculated as 18.7 µM at 24 h and 9.4 µM at 48 h, whereas the corresponding values in HaCaT cells were 28.3 µM and 19.6 µM, respectively (Figure 1C,D). In both treatment groups, prolonged exposure enhanced the cytotoxic effects of the tested compounds, particularly in OVCAR3 cells. Vehicle control treatment with 0.1% DMSO did not significantly affect cell viability in either cell line.

### 2.2. Synergistic Cytotoxic Effects of Combined RA and Gem Treatment

**Figure 2:**
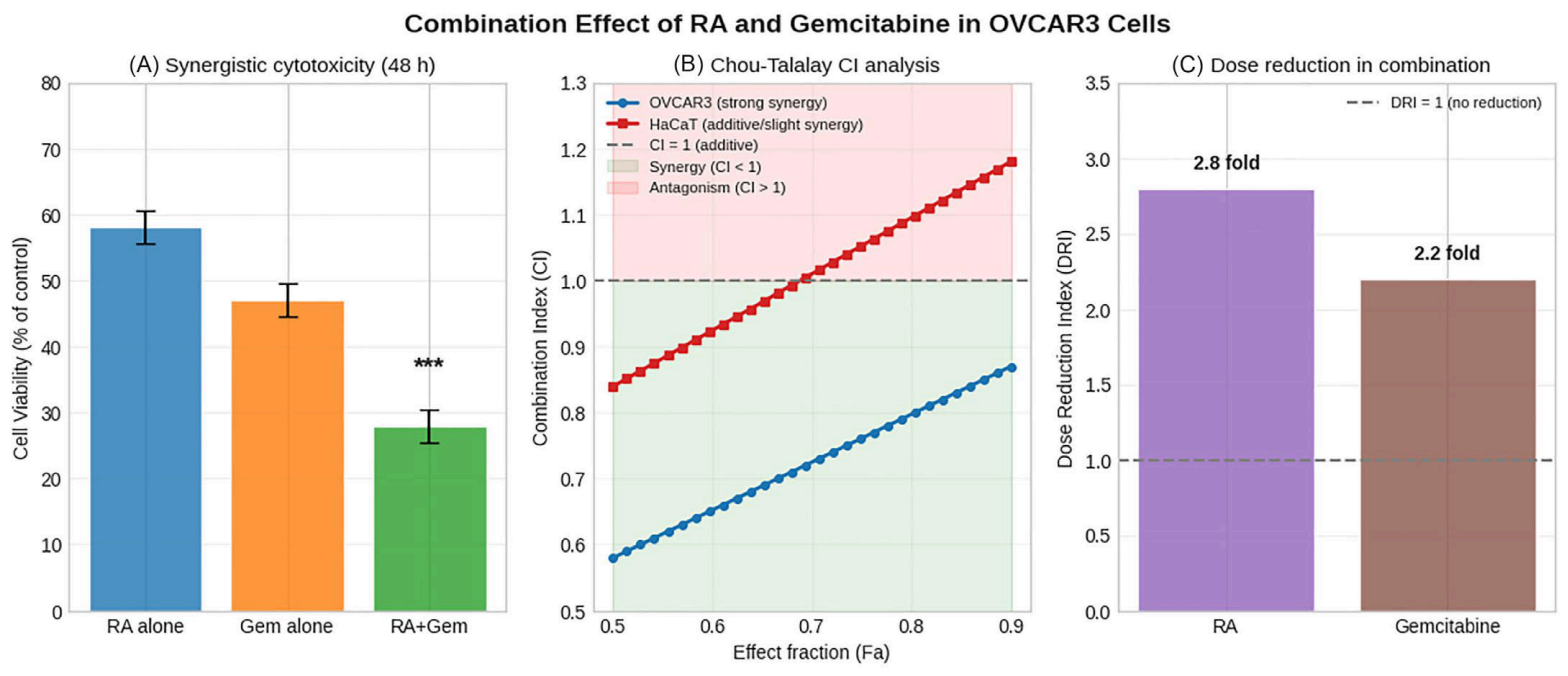
Evaluation of the combined effects of RA and Gem in OVCAR3 and HaCaT cells. (**A**) Cell viability following 48 h treatment with RA and Gem administered individually or in combination at their respective 48 h IC_50_ concentrations. The concentrations used were 98.4 µM RA and 9.4 µM Gem for OVCAR3 cells, and 198.3 µM RA and 19.6 µM Gem for HaCaT cells. (**B**) CI analysis of RA and Gem in OVCAR3 and HaCaT cells according to the Chou–Talalay method across different Fa levels. (**C**) DRI analysis demonstrating the dose-reduction potential of RA and Gem under combination treatment conditions. Data are presented as mean ± SD (*n* = 3). Statistical analysis for panel (**A**) was performed using one-way ANOVA followed by Tukey’s post hoc test. *** *p* < 0.001 versus both single-agent treatment groups (RA alone and Gem alone).

To evaluate the combined effects of RA and Gem, both compounds were administered using a fixed-ratio combination design. Combined treatment significantly reduced OVCAR3 cell viability compared with single-agent treatments following 48 h exposure (*p* < 0.001; Figure 2A). The RA + Gem combination produced a greater reduction in viability than either RA or Gem administered alone under the tested experimental conditions.

Drug interaction analysis was performed using the Chou–Talalay method. In OVCAR3 cells, CI values ranged between 0.58 and 0.87 across effect fraction (Fa) levels of 0.50–0.90, indicating synergistic interaction between RA and Gem (CI < 1; Figure 2B). In contrast, HaCaT cells exhibited CI values ranging from 0.84 to 1.18 under the same treatment conditions, suggesting responses varying from additive to mildly synergistic effects depending on the effect level.

Drug reduction index (DRI) analysis demonstrated that combination treatment enabled dose reductions of approximately 2.8-fold for RA and 2.2-fold for Gem while maintaining comparable cytotoxic effects in OVCAR3 cells (Figure 2C). These findings support enhanced cytotoxic activity of the RA + Gem combination in ovarian cancer cells compared with single-agent treatment conditions.

### 2.3. Effects of Combined RA and Gem Treatment on Apoptosis

**Figure 3:**
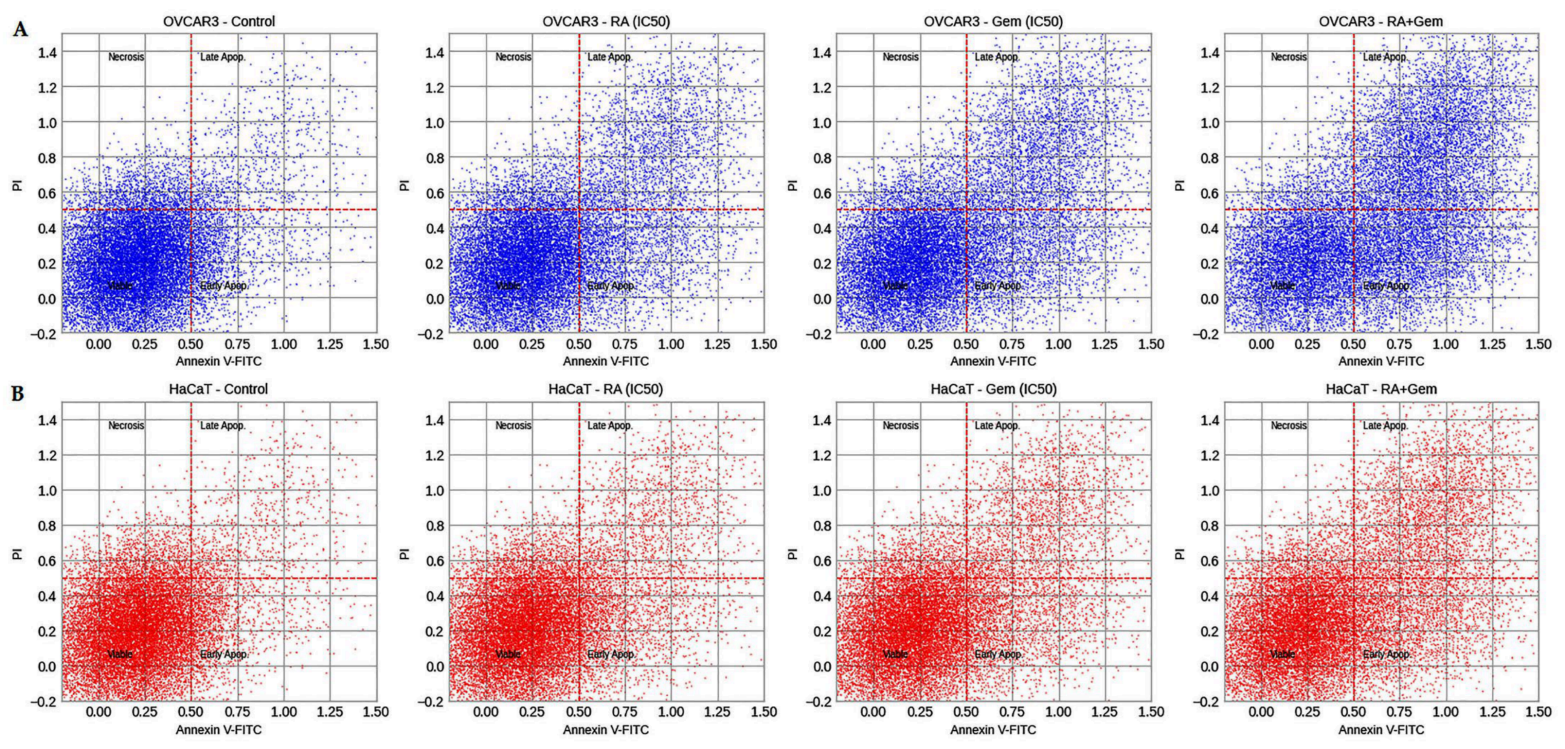
Representative Annexin V-FITC/PI flow cytometry dot plots of OVCAR3 and HaCaT cells following 48 h treatment with RA, Gem, or the RA + Gem combination at their respective IC_50_ concentrations. Apoptotic cell populations were analyzed by Annexin V-FITC/PI double staining. Quadrants represent viable (Annexin V^−^/PI^−^), early apoptotic (Annexin V^+^/PI^−^), late apoptotic (Annexin V^+^/PI^+^), and necrotic (Annexin V^−^/PI^+^) cell populations. (**A**) OVCAR3 cells. (**B**) HaCaT cells. The red dashed lines indicate the gating thresholds used to define the four quadrants.

**Figure 4:**
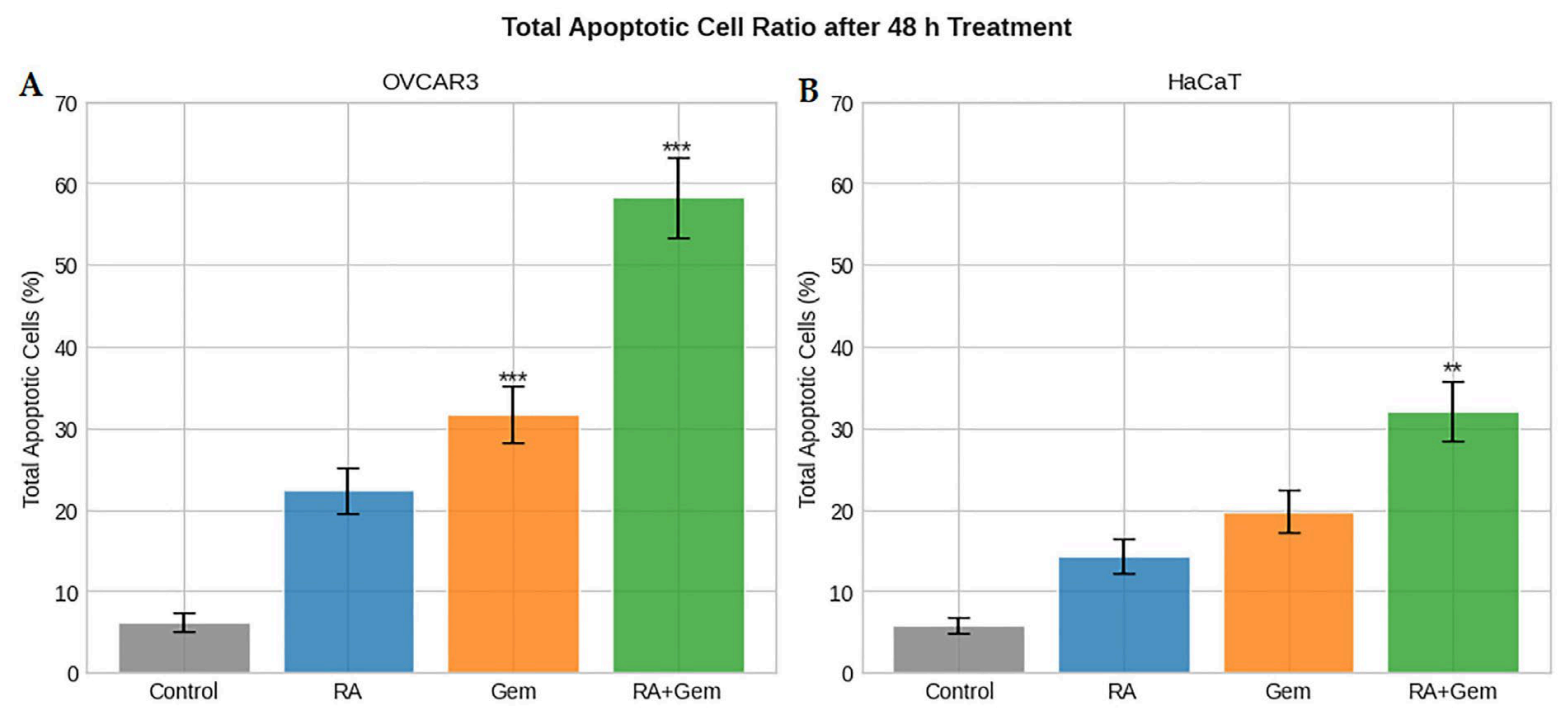
Quantification of total apoptotic cell populations following 48 h treatment with RA, Gem, or combined RA + Gem treatment. Total apoptotic cells were calculated as the sum of early and late apoptotic populations determined by Annexin V-FITC/PI flow cytometry. (**A**) OVCAR3 cells. (**B**) HaCaT cells. Data are presented as mean ± SD (*n* = 3). In OVCAR3 cells, Gem and RA + Gem treatments significantly increased total apoptosis compared with the control group (*** *p* < 0.001). In HaCaT cells, RA + Gem treatment significantly increased total apoptosis compared with the control group (** *p* < 0.01).

Apoptotic cell death following RA, Gem, and combined RA + Gem treatment was evaluated by Annexin V-FITC/PI double staining after 48 h exposure. Representative flow cytometry dot plots are presented in Figure 3. In OVCAR3 cells, the total apoptotic cell population in the control group was 6.2 ± 1.2%, whereas treatment with RA or Gem at their respective IC_50_ concentrations increased apoptotic rates to 22.4 ± 2.8% and 31.7 ± 3.4%, respectively (Figure 4A). Combined RA + Gem treatment further increased the total apoptotic population to 58.3 ± 4.9%, representing a statistically significant increase compared with both single-agent treatment groups (*p* < 0.001; Figure 4A). In addition to the increase in total apoptosis, combined treatment markedly elevated both early apoptotic (Annexin V^+^/PI^−^) and late apoptotic (Annexin V^+^/PI^+^) cell populations compared with the control and single-agent groups.

In HaCaT cells, the total apoptotic population was 5.8 ± 0.9% in the control group and increased to 14.3 ± 2.1%, 19.8 ± 2.6%, and 32.1 ± 3.7% following RA, Gem, and combined RA + Gem treatment, respectively (Figure 4B). Although combined treatment also increased apoptosis in HaCaT cells, the apoptotic response remained comparatively lower than that observed in OVCAR3 cells under the same experimental conditions. These findings suggest greater sensitivity of OVCAR3 cells to the combined treatment regimen.

### 2.4. Effects of Combined RA and Gem Treatment on Cell Cycle Progression

**Figure 5:**
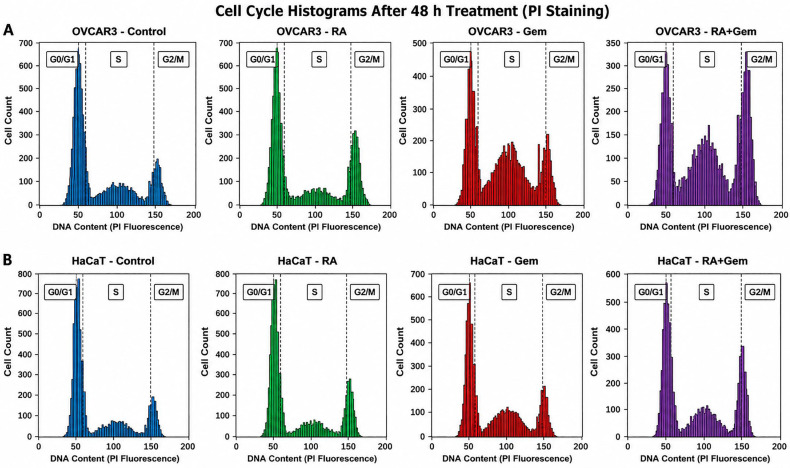
Representative cell cycle histograms obtained by PI staining following 48 h treatment with RA, Gem, or the RA + Gem combination. DNA content was analyzed by flow cytometry to evaluate cell cycle distribution. (**A**) OVCAR3 cells. (**B**) HaCaT cells. Histograms show the distribution of cell populations across the G0/G1, S, and G2/M phases.

**Figure 6:**
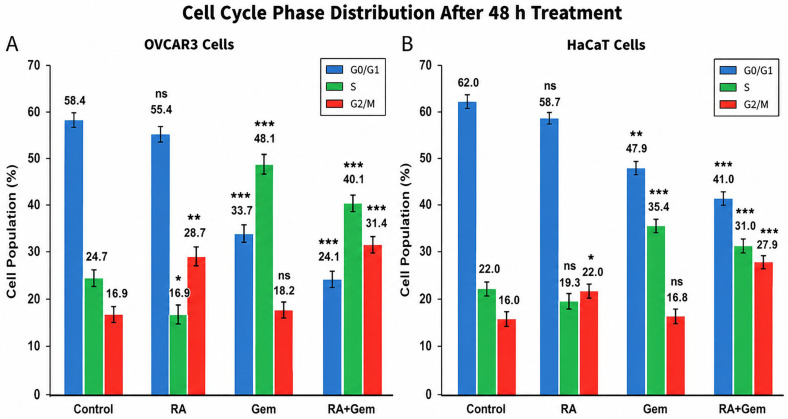
Quantitative analysis of cell cycle phase distribution following 48 h treatment with RA, Gem, or the RA + Gem combination. Cell cycle distribution was determined by flow cytometric analysis of PI-stained cells and expressed as the percentage of cells in the G0/G1, S, and G2/M phases. (**A**) OVCAR3 cells. (**B**) HaCaT cells. Data are presented as mean ± SD (*n* = 3). Statistical significance was analyzed using one-way ANOVA followed by Tukey’s multiple-comparison post hoc test. * *p* < 0.05, ** *p* < 0.01, *** *p* < 0.001 versus the control group; ns, not significant.

Cell cycle distribution following RA, Gem, and combined RA + Gem treatment was evaluated by PI staining after 48 h exposure. Representative DNA content histograms and quantitative phase distributions are presented in Figure 5 and Figure 6, respectively.

In OVCAR3 cells, the control group exhibited cell population distributions of 58.4 ± 3.2% in G0/G1 phase, 24.7 ± 2.1% in S phase, and 16.9 ± 1.8% in G2/M phase (Figure 5A and Figure 6A). RA treatment increased the proportion of cells in the G2/M phase to 28.3 ± 2.6%, whereas Gem treatment resulted in marked accumulation in the S phase (48.1 ± 3.2%). Combined RA + Gem treatment induced redistribution of the cell population across both S and G2/M phases, with S-phase and G2/M populations increasing to 41.3 ± 3.0% and 30.4 ± 3.1%, respectively, accompanied by a reduction in the G0/G1 population to 28.3 ± 2.5% (*p* < 0.001 vs. control).

In HaCaT cells, treatment-associated alterations in cell cycle distribution were also observed following RA, Gem, and combined treatment exposure; however, these changes were comparatively less pronounced than those detected in OVCAR3 cells (Figure 5B and Figure 6B). The combined treatment group demonstrated moderate redistribution across S and G2/M phases without the marked phase accumulation observed in ovarian cancer cells.

### 2.5. Effects of Combined RA and Gem Treatment on Intracellular ROS Accumulation

**Figure 7:**
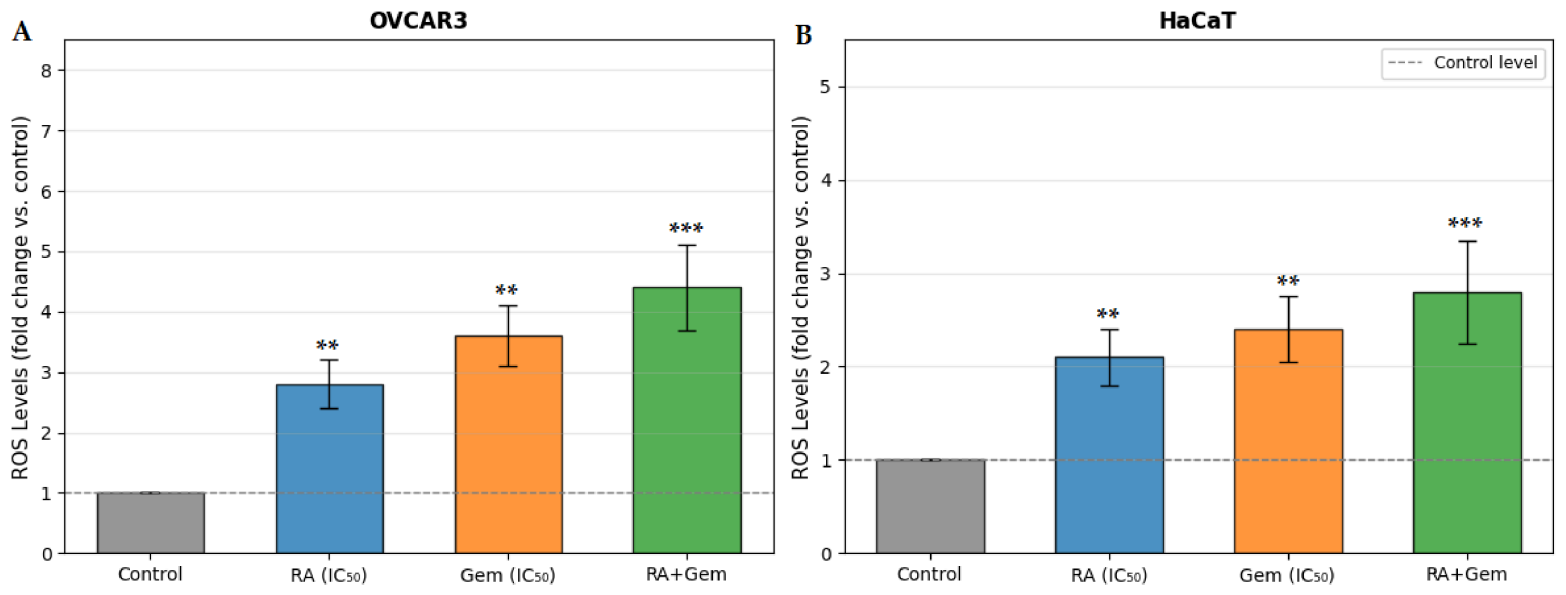
Intracellular ROS levels following 48 h treatment with RA, Gem, or the RA + Gem combination determined using DCFH-DA staining followed by flow cytometric analysis. ROS levels were expressed relative to untreated control cells. (**A**) OVCAR3 cells. (**B**) HaCaT cells. Data are presented as mean ± SD (*n* = 3). ** *p* < 0.01 and *** *p* < 0.001 versus the control group.

Intracellular ROS levels following RA, Gem, and combined RA + Gem treatment were evaluated using DCFH-DA staining after 48 h exposure. In OVCAR3 cells, RA and Gem treatment at their respective IC_50_ concentrations increased ROS levels by approximately 2.8 ± 0.4-fold and 3.6 ± 0.5-fold relative to the control group, respectively (Figure 7A). Combined RA + Gem treatment further elevated intracellular ROS accumulation to approximately 4.4 ± 0.7-fold compared with untreated control cells (*p* < 0.001).

In HaCaT cells, treatment-associated increases in intracellular ROS levels were also observed following RA, Gem, and combined treatment exposure; however, these increases remained comparatively lower than those detected in OVCAR3 cells. RA and Gem treatment increased ROS levels by approximately 2.1 ± 0.3-fold and 2.4 ± 0.4-fold, respectively, whereas combined RA + Gem treatment resulted in an approximately 2.8 ± 0.6-fold increase relative to the control group (Figure 7B). These findings suggest enhanced oxidative stress-associated responses following combined treatment, particularly in ovarian cancer cells.

### 2.6. Effects of NAC Pretreatment on ROS Accumulation and Cytotoxicity

To evaluate the contribution of oxidative stress-associated mechanisms to the cytotoxic effects induced by combined RA + Gem treatment, NAC pretreatment experiments were performed in OVCAR3 cells. Cells were pretreated with NAC (5 mM) prior to 48 h exposure to RA, Gem, or combined RA + Gem treatment.

**Figure 8:**
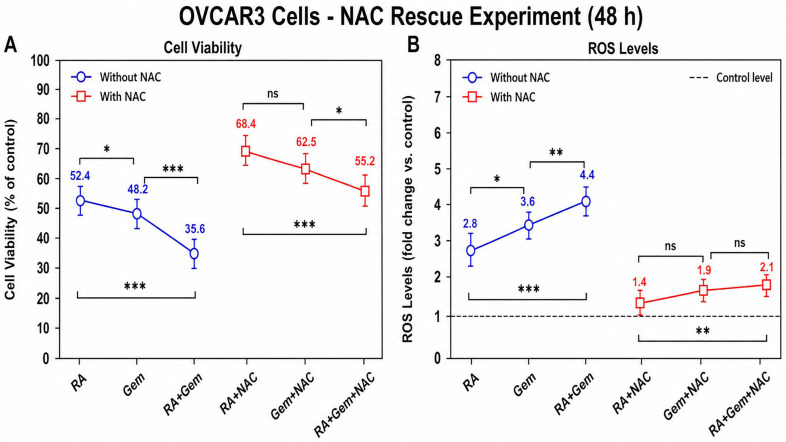
Effects of NAC pretreatment on cell viability and intracellular ROS levels in OVCAR3 cells following 48 h treatment with RA, Gem, or the RA + Gem combination. Cells were pretreated with NAC (5 mM) prior to treatment. Cell viability was assessed using the MTT assay, and intracellular ROS levels were determined by DCFH-DA staining followed by flow cytometric analysis. (**A**) Cell viability. (**B**) Intracellular ROS levels. Numerical values are shown above the data points. Data are presented as mean ± SD (*n* = 3). Statistical significance is indicated in the figure. * *p* < 0.05, ** *p* < 0.01, *** *p* < 0.001; ns, not significant.

Combined RA + Gem treatment markedly increased intracellular ROS accumulation in OVCAR3 cells (Figure 8B). NAC pretreatment attenuated treatment-associated ROS generation, resulting in substantially lower ROS levels compared with the corresponding treatment groups without NAC.

Similarly, combined RA + Gem treatment reduced cell viability, whereas NAC pretreatment partially restored cell viability under the same treatment conditions (Figure 8A). NAC treatment alone did not markedly affect either ROS levels or cell viability.

These findings suggest that ROS accumulation contributes, at least in part, to the cytotoxic effects induced by combined RA + Gem treatment. However, the incomplete restoration of cell viability following NAC pretreatment indicates that oxidative stress is unlikely to be the sole mechanism underlying the observed antitumor response.

### 2.7. Effects of Combined RA and Gem Treatment on Apoptosis- and Proliferation-Related Gene Expression

**Figure 9:**
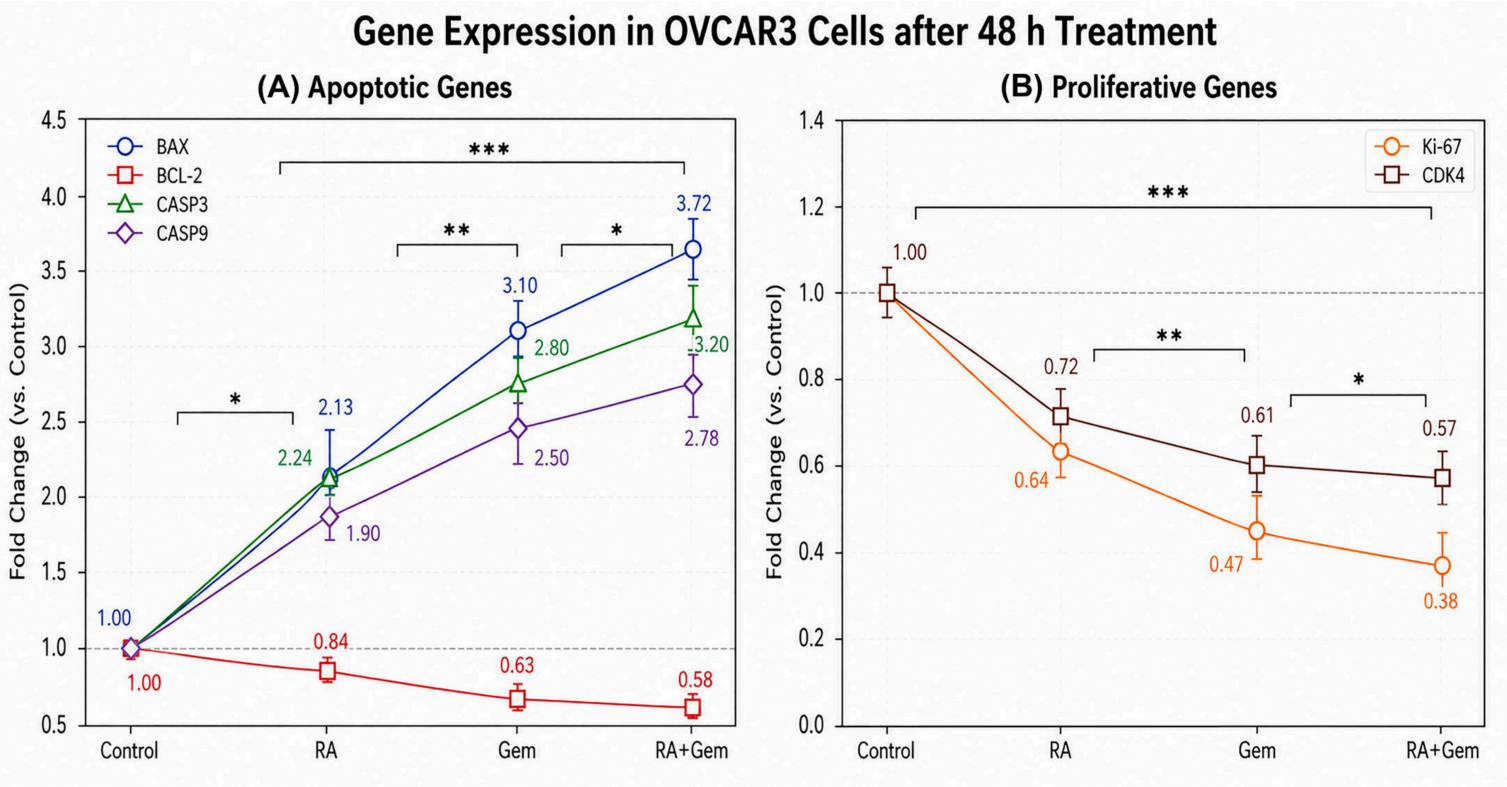
Effects of RA, Gem, and the RA + Gem combination on apoptosis- and proliferation-related gene expression in OVCAR3 cells following 48 h treatment. Gene expression levels were evaluated by RT-qPCR and normalized relative to the untreated control cells using the 2^−ΔΔCt^ method. Numerical fold-change values are displayed adjacent to the corresponding data points. Data are presented as mean ± SD (*n* = 3). Statistical significance for the indicated comparisons is shown in the figure. (**A**) Expression profiles of the apoptosis-related genes *BAX*, *BCL2*, *CASP3*, and *CASP9*. (**B**) Expression profiles of the proliferation-related genes *MKI67* and *CDK4*. * *p* < 0.05, ** *p* < 0.01, and *** *p* < 0.001 for the indicated comparisons.

The effects of RA, Gem, and combined RA + Gem treatment on apoptosis- and proliferation-related gene expression in OVCAR3 cells were evaluated by RT-qPCR following 48 h exposure (Figure 9).

Combined RA + Gem treatment markedly increased the expression levels of the proapoptotic genes *BAX*, *CASP3*, and *CASP9* compared with untreated control cells (Figure 9A). Relative to the control group, combined treatment increased *BAX*, *CASP3*, and *CASP9* expression by approximately 3.7 ± 0.4-fold, 3.2 ± 0.5-fold, and 2.8 ± 0.3-fold, respectively (*p* < 0.001). RA treatment alone increased *BAX*, *CASP3*, and *CASP9* expression by approximately 2.1 ± 0.25-fold, 2.2 ± 0.30-fold, and 1.9 ± 0.25-fold, respectively, whereas Gem treatment increased expression of the same genes by approximately 3.1 ± 0.30-fold, 2.8 ± 0.35-fold, and 2.5 ± 0.30-fold.

In contrast, combined treatment reduced expression of the antiapoptotic gene *BCL2* to approximately 0.58 ± 0.04-fold relative to control cells (*p* < 0.001). RA and Gem treatment alone reduced *BCL2* expression to approximately 0.85 ± 0.08-fold and 0.62 ± 0.06-fold, respectively. In addition, the *BAX*/*BCL2* ratio was elevated in the combined treatment group compared with single-agent treatment groups, consistent with a proapoptotic gene expression profile.

Expression levels of the proliferation-related genes *MKI67* and *CDK4* were also reduced following treatment exposure (Figure 9B). Combined RA + Gem treatment decreased *MKI67* expression to approximately 0.38 ± 0.03-fold and *CDK4* expression to approximately 0.58 ± 0.04-fold relative to control cells (*p* < 0.001). RA and Gem single-agent treatments also reduced expression of both genes, although the most pronounced suppression was observed following combined treatment.

### 2.8. Immunocytochemical Evaluation of Caspase-3 Activation and Nuclear Morphology

**Figure 10:**
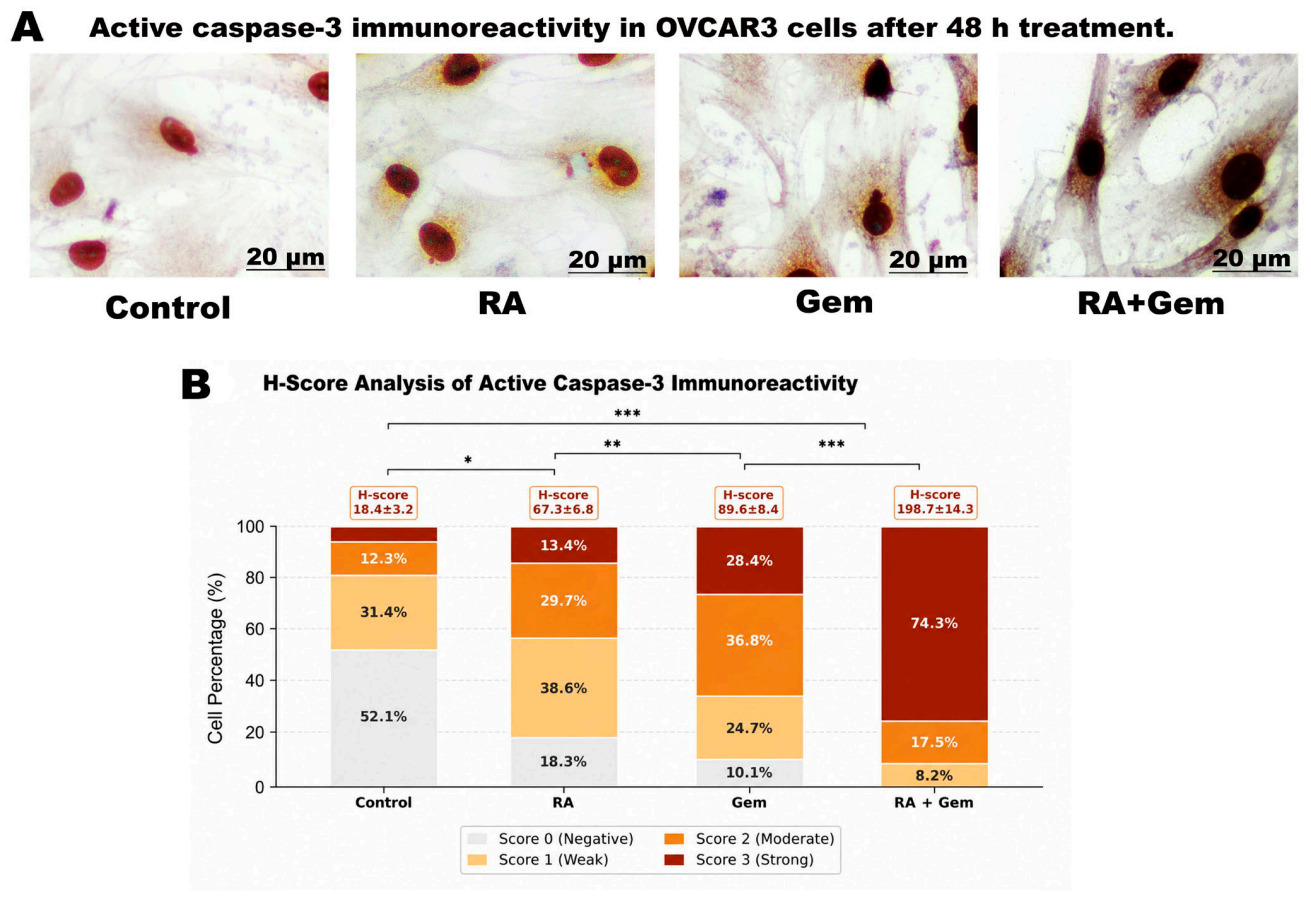
Representative immunocytochemical evaluation and semiquantitative analysis of active caspase-3 immunoreactivity in OVCAR3 cells following 48 h treatment with RA, Gem, or combined RA + Gem treatment. (**A**) Representative immunocytochemical images of active caspase-3 staining. Active caspase-3 immunoreactivity was visualized using DAB chromogen with hematoxylin counterstaining. Brown cytoplasmic staining indicates positive active caspase-3 immunoreactivity. Scale bar = 20 µm. Original magnification: ×200. (**B**) H-score analysis and staining intensity distribution of active caspase-3 immunoreactivity. Staining intensity was categorized as Score 0 (negative), Score 1 (weak), Score 2 (moderate), and Score 3 (strong). H-score values are presented above each column. Data are presented as mean ± SD (*n* = 3). Statistical comparisons are indicated in the figure. * *p* < 0.05, ** *p* < 0.01, *** *p* < 0.001. Interrater reliability was assessed using intraclass correlation coefficient (ICC) analysis.

Active caspase-3 immunoreactivity in OVCAR3 cells following 48 h treatment with RA, Gem, or combined RA + Gem treatment was evaluated by immunocytochemistry using DAB chromogen staining and semiquantitative H-score analysis (Figure 10). Representative immunocytochemical images (Figure 10A) showed weak cytoplasmic brown DAB staining in the control group, whereas RA and Gem treatment increased active caspase-3 immunoreactivity. The strongest staining intensity was observed in the combined RA + Gem treatment group.

Semiquantitative H-score analysis (Figure 10B) supported these observations. The control group exhibited an H-score of 18.4 ± 3.2, which increased significantly following RA (67.3 ± 6.8, *p* < 0.05 vs. control) and Gem (89.6 ± 8.4, *p* < 0.01 vs. RA) treatment. The combined RA + Gem treatment produced the highest H-score (198.7 ± 14.3), representing a significant increase compared with both the control and Gem-treated groups (** *p* < 0.001).

Analysis of staining intensity further demonstrated a progressive shift toward stronger active caspase-3 expression following treatment. In the control group, most cells were negative for active caspase-3 staining (Score 0, 52.1%), whereas the proportion of strongly positive cells (Score 3) increased from 4.2% in the control group to 13.4% following RA treatment, 28.4% following Gem treatment, and 74.3% following combined RA + Gem treatment. Concurrently, the proportion of negatively stained cells decreased to 18.3%, 10.1%, and 8.2%, respectively.

**Figure 11:**
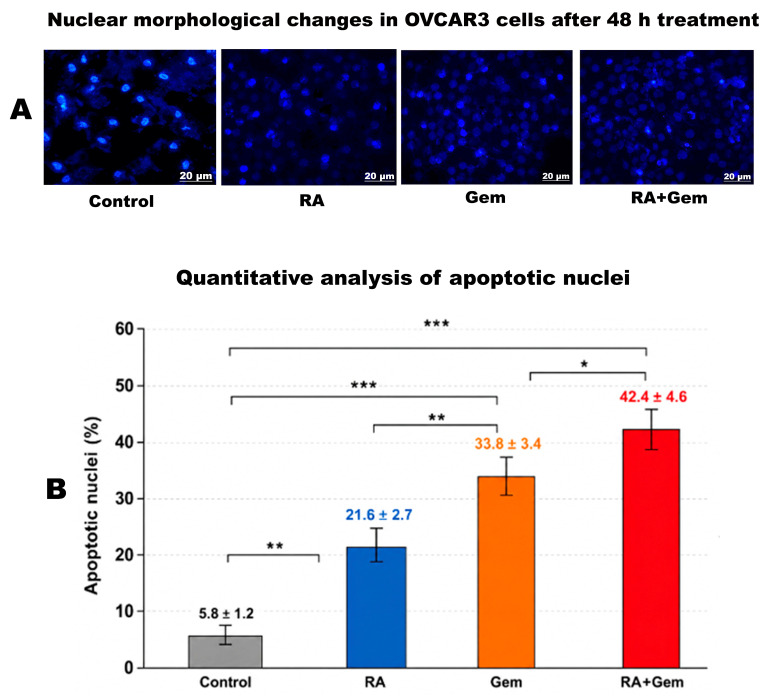
Nuclear morphological changes and quantitative analysis of apoptotic nuclei in OVCAR3 cells following 48 h treatment with RA, Gem, or combined RA + Gem treatment. (**A**) Representative fluorescence microscopy images of OVCAR3 cells stained with NucBlue Live ReadyProbes Reagent (Hoechst 33342) to evaluate nuclear morphology. Blue fluorescence represents Hoechst 33342-stained nuclei. Scale bar = 20 µm. Original magnification: ×200. (**B**) Quantitative analysis of apoptotic nuclei expressed as the percentage of condensed and/or fragmented nuclei relative to the total number of Hoechst 33342-stained nuclei. At least 300 nuclei were evaluated from five randomly selected microscopic fields for each treatment group. Data are presented as mean ± SD (*n* = 3). Statistical analysis was performed using one-way ANOVA followed by Tukey’s post hoc test. ** *p* < 0.01 for Control versus RA; *** *p* < 0.001 for Control versus Gem; ** *p* < 0.01 for RA versus Gem; * *p* < 0.05 for Gem versus RA + Gem; and *** *p* < 0.001 for Control versus RA + Gem, as indicated.

Nuclear morphology was further evaluated using NucBlue (Hoechst 33342) fluorescence staining following treatment exposure (Figure 11A). Control cells exhibited morphologically intact nuclei with relatively homogeneous chromatin distribution. In contrast, RA- and Gem-treated cells demonstrated nuclear condensation and irregular chromatin organization. The combined RA + Gem treatment group exhibited more pronounced nuclear morphological alterations, including chromatin condensation and nuclear fragmentation, consistent with apoptotic cell death.

Quantitative analysis of DAPI-stained nuclei confirmed the morphological observations (Figure 11B). The percentage of apoptotic nuclei increased from 5.8 ± 1.2% in the control group to 21.6 ± 2.7% following RA treatment and 33.8 ± 3.4% following Gem treatment. Combined RA + Gem treatment produced the highest proportion of apoptotic nuclei (42.4 ± 4.6%), which was significantly higher than both the control and single-agent treatment groups (*p* < 0.001).

### 2.9. Enhanced Antitumor Activity of RA–Gem Combination in 3D OVCAR3 Tumor Spheroids

**Figure 12:**
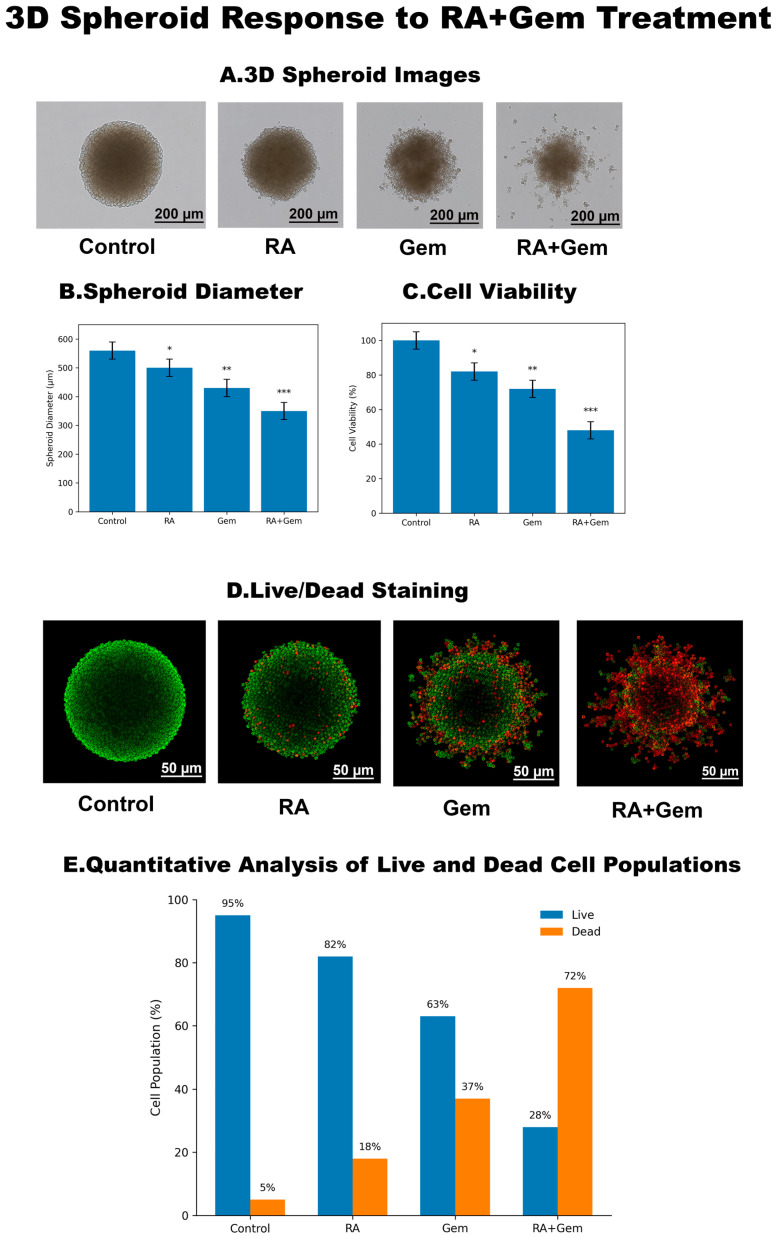
Effects of RA, Gem, and combined RA + Gem treatment on 3D OVCAR3 tumor spheroids following 48 h treatment. (**A**) Representative bright-field images of spheroids treated with vehicle control, RA, Gem, or combined RA + Gem. Scale bar = 200 µm. (**B**) Quantitative analysis of spheroid diameter. (**C**) ATP-based quantification of spheroid viability. (**D**) Representative live/dead fluorescence images of spheroids stained with Calcein-AM (green, viable cells) and Ethidium Homodimer-1 (red, dead cells). Scale bar = 50 µm. (**E**) Quantitative analysis of live and dead cell populations. Data are presented as mean ± SD (*n* = 3). Statistical analysis was performed using one-way ANOVA followed by Tukey’s post hoc test (* *p* < 0.05, ** *p* < 0.01, *** *p* < 0.001 versus control).

Under control conditions, OVCAR3 cells formed compact and structurally well-organized 3D spheroids with smooth borders and preserved architectural integrity. RA treatment induced moderate morphological alterations characterized by mild reduction in spheroid compactness and early peripheral irregularities while largely maintaining overall spheroid structure. In contrast, Gem treatment produced more pronounced architectural disruption, including decreased compactness, irregular spheroid borders, and partial fragmentation. The combined RA + Gem treatment resulted in the most extensive structural deterioration, characterized by marked spheroid shrinkage, severe loss of compact organization, widespread cellular dispersion, and advanced fragmentation, consistent with substantial impairment of spheroid integrity (Figure 12A).

Quantitative analysis of spheroid diameter supported these morphological observations (Figure 12B). Compared with control spheroids (approximately 560 µm), RA treatment moderately reduced spheroid diameter, whereas Gem treatment induced a more substantial reduction. Importantly, the RA + Gem combination produced the greatest decrease in spheroid size (approximately 350 µm), significantly lower than both single-agent treatment groups (*p* < 0.01–0.001), indicating enhanced growth-inhibitory activity under 3D culture conditions.

Assessment of ATP-based spheroid viability demonstrated that both RA and Gem monotherapies significantly reduced viability relative to control spheroids (Figure 12C). RA treatment decreased viability to approximately 82%, whereas Gem reduced viability to nearly 68%. Notably, the combination group exhibited the strongest cytotoxic response, reducing viability to approximately 48% (*p* < 0.001 vs. single-agent groups), consistent with preservation of enhanced antitumor activity within the 3D tumor model.

Live/dead fluorescence imaging further confirmed treatment-associated cytotoxicity (Figure 12D). Control spheroids displayed intense and homogeneous green fluorescence with minimal red signal, indicating high cellular viability and preserved spheroid architecture. RA-treated spheroids exhibited a mild increase in red fluorescence accompanied by limited peripheral disorganization, consistent with moderate induction of cell death. Gem-treated spheroids demonstrated substantially increased red fluorescence together with more evident architectural disruption and reduced compactness. In contrast, RA + Gem-treated spheroids exhibited extensive red fluorescence dominance, marked reduction in green signal intensity, and severe spheroid fragmentation, indicating widespread loss of viability and profound disruption of spheroid organization.

Quantitative analysis of live and dead cell populations paralleled fluorescence imaging findings (Figure 12E). Control spheroids contained predominantly viable cells (~95% live cells), whereas RA and Gem treatments progressively increased the proportion of non-viable cells. The RA + Gem combination produced the highest dead-cell fraction (~72%) together with the lowest proportion of viable cells (~28%), supporting the marked cytotoxic effect observed microscopically. Because fluorescence intensity and signal distribution may vary within dense 3D spheroid structures, live/dead imaging results were interpreted qualitatively alongside quantitative viability analyses.

Taken together, these findings demonstrate that the enhanced antitumor activity observed in monolayer cultures is preserved in a 3D ovarian cancer model and is associated with increased cytotoxic and growth-inhibitory effects under physiologically more relevant conditions, rather than definitive evidence of a specific molecular mechanism.

### 2.10. ROS Contributes to, but Does Not Fully Mediate, RA–Gem-Induced Cytotoxicity in 3D OVCAR3 Spheroids

**Figure 13:**
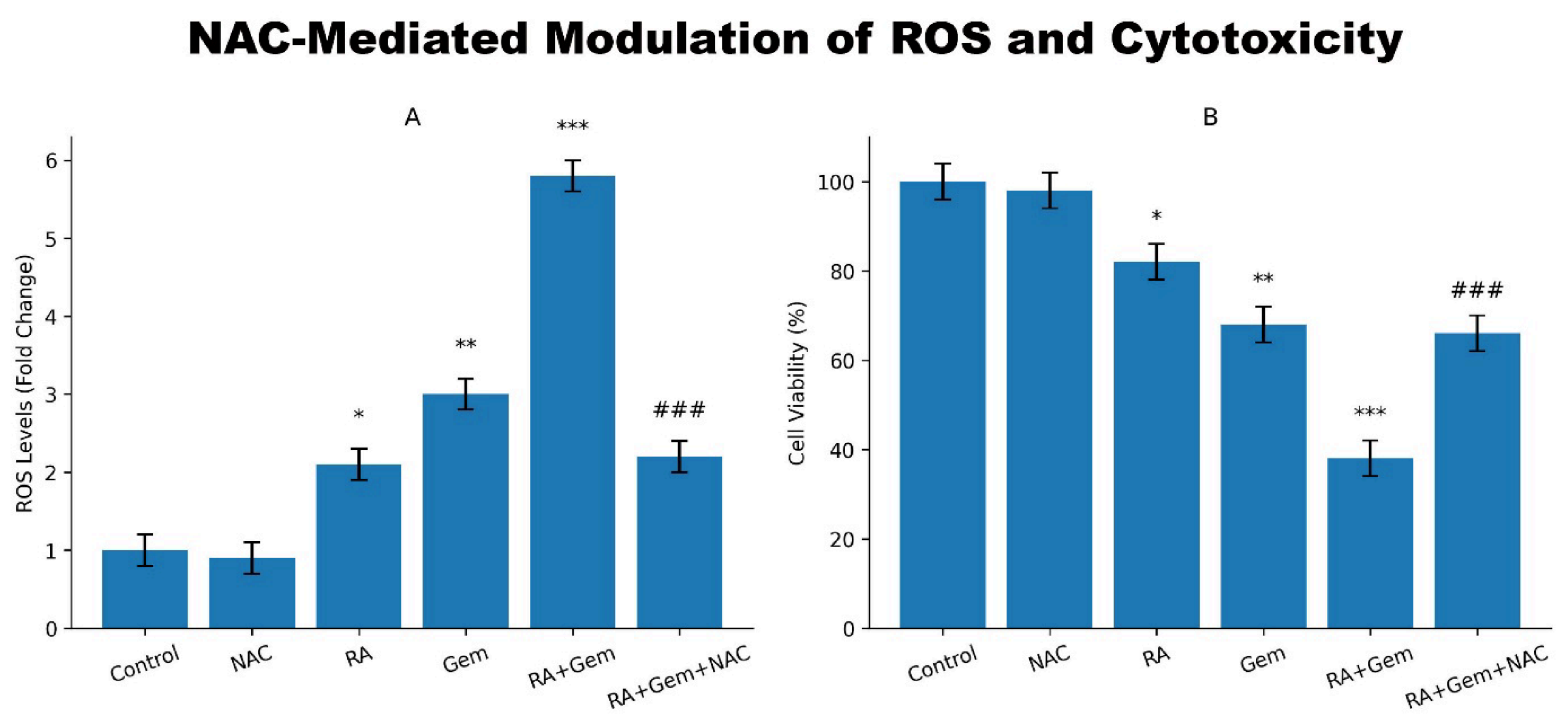
Effects of NAC pretreatment on intracellular ROS levels and cell viability in OVCAR3 spheroids following treatment with RA, Gem, or the RA + Gem combination. (**A**) Intracellular ROS levels in OVCAR3 spheroids measured after treatment with RA, Gem, or the RA + Gem combination in the presence or absence of NAC pretreatment. (**B**) Cell viability of OVCAR3 spheroids following treatment with RA, Gem, or the RA + Gem combination in the presence or absence of NAC pretreatment. Data are presented as mean ± SD (*n* = 3). Statistical analysis was performed using one-way ANOVA followed by Tukey’s post hoc test (* *p* < 0.05, ** *p* < 0.01, *** *p* < 0.001 versus control; ### *p* < 0.001 versus the RA + Gem group).

To evaluate the functional contribution of oxidative stress to treatment-associated cytotoxicity, intracellular ROS levels and spheroid viability were assessed following NAC pretreatment (Figure 13A,B).

ROS analysis demonstrated low basal oxidative stress levels in control and NAC-only groups (Figure 13A). RA treatment moderately increased intracellular ROS accumulation (~2.1-fold), whereas Gem treatment induced a more pronounced elevation (~3.0-fold). Notably, the RA + Gem combination produced the highest ROS accumulation (~5.8-fold vs. control; *p* < 0.001), indicating enhanced oxidative stress under dual-treatment conditions. Pretreatment with NAC markedly attenuated ROS levels in the RA + Gem group, reducing ROS accumulation to nearly 2.2-fold above control levels (### *p* < 0.001 vs. RA + Gem), confirming effective suppression of oxidative stress.

Consistent with ROS modulation, viability analysis demonstrated that control and NAC-only spheroids maintained high viability, whereas RA and Gem treatments reduced spheroid viability to approximately 82% and 68%, respectively (Figure 13B). The RA + Gem combination produced the strongest cytotoxic effect, reducing viability to nearly 38% of control levels (*p* < 0.001). Importantly, NAC pretreatment partially restored spheroid viability in the combination group to approximately 66% (### *p* < 0.001 vs. RA + Gem alone), indicating a functional rescue effect.

However, viability in NAC-pretreated spheroids remained substantially lower than untreated control levels, demonstrating that ROS scavenging only partially reverses treatment-associated cytotoxicity. Importantly, these findings indicate that ROS generation functionally contributes to the cytotoxic effects associated with the RA–Gem combination in 3D ovarian cancer spheroids, but does not fully account for the observed antitumor activity. These results therefore suggest the involvement of additional mechanisms, potentially including mitochondrial apoptotic signaling and cell cycle-associated responses, rather than ROS acting as the sole mediator of treatment-induced cytotoxicity.

### 2.11. Bioinformatic Findings

#### 2.11.1. Protein–Protein Interaction Network Analysis

**Figure 14:**
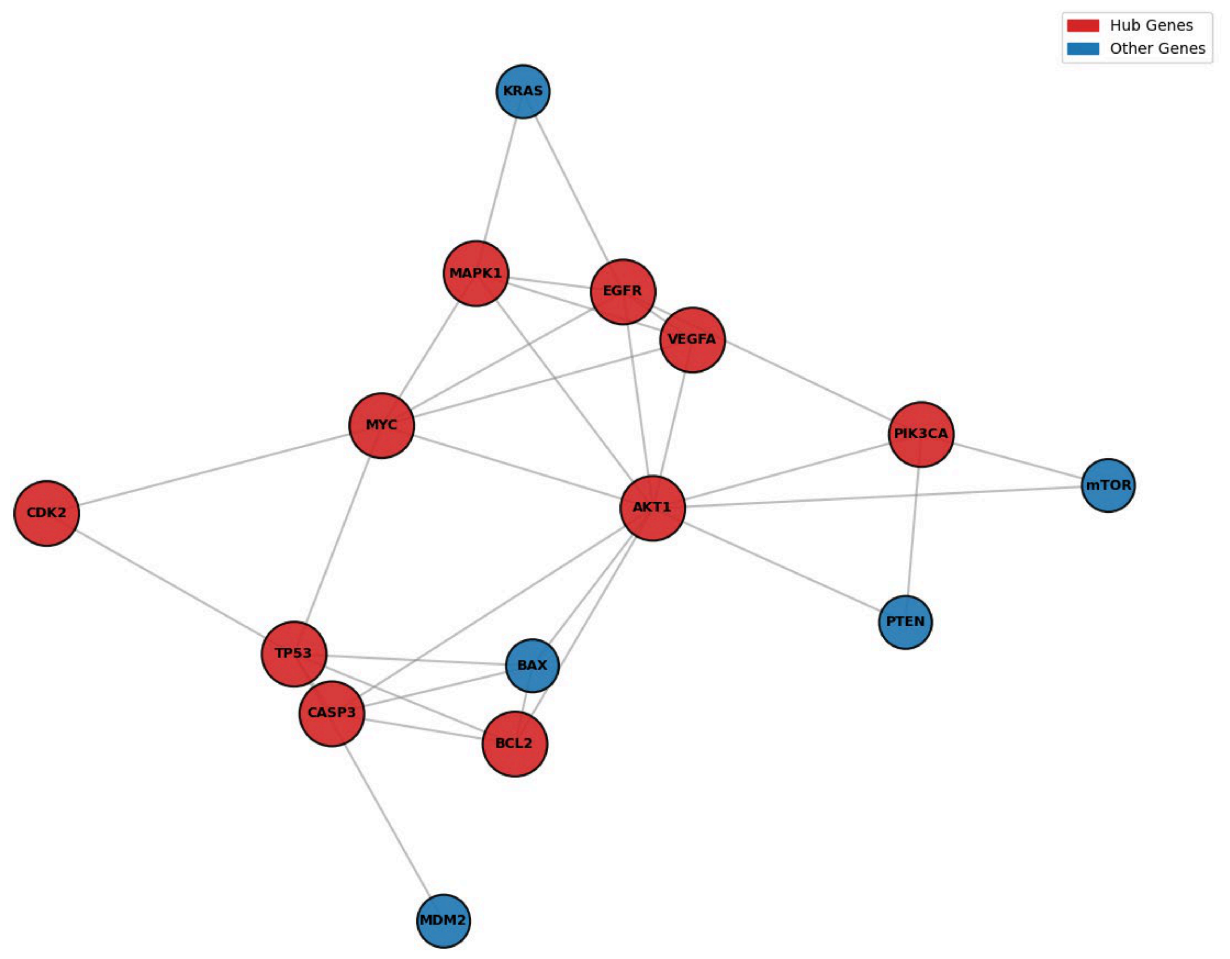
Protein–protein interaction (PPI) network generated using the STRING database (version 11.5; confidence score ≥ 0.700) and visualized in Cytoscape (version 3.10.0). Nodes represent genes/proteins included in the network, whereas edges indicate known or predicted functional associations identified by STRING. Red nodes indicate genes with relatively higher interaction connectivity within the generated network, whereas blue nodes represent additional network-associated genes. The network is presented as an exploratory bioinformatic analysis to illustrate potential functional relationships among the investigated genes.

To further explore potential functional associations among apoptosis- and proliferation-related genes affected by treatment, a protein–protein interaction (PPI) network was constructed using the STRING database and visualized in Cytoscape software (version 3.10.0) (Figure 14). The generated network demonstrated extensive predicted interactions among genes associated with apoptosis, proliferation, and survival-related signaling pathways.

**Figure 15:**
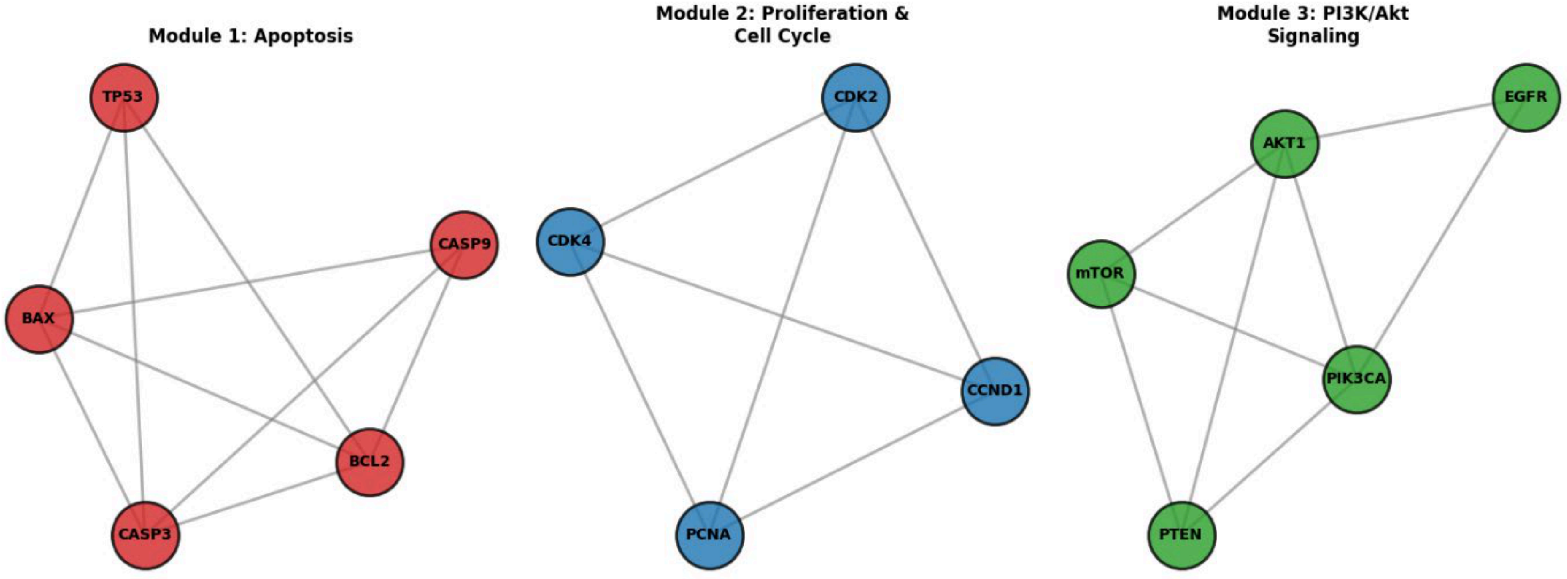
Functional cluster analysis of the protein–protein interaction network performed using the Molecular Complex Detection (MCODE) algorithm in Cytoscape. Three major functional clusters were identified, including apoptosis-related genes (*TP53*, *BCL2*, *CASP3*, *BAX*, and *CASP9*), proliferation- and cell cycle-associated genes (*CDK2*, *CCND1*, *PCNA*, *MKI67*, and *CDK4*), and PI3K/Akt-associated signaling genes (*AKT1*, *PIK3CA*, *PTEN*, *MTOR*, and *EGFR*). MCODE cluster score threshold was set to ≥3. The identified clusters represent predicted functional groupings derived from bioinformatic network analysis and are presented for exploratory purposes only.

Module analysis performed using the Molecular Complex Detection (MCODE) algorithm revealed three major functional clusters within the interaction network (Figure 15). The first cluster was primarily associated with apoptosis-related genes, including *TP53*, *BCL2*, *CASP3*, *BAX*, and *CASP9*. The second cluster included proliferation- and cell cycle-associated genes such as *CDK2*, *CCND1*, *PCNA*, *MKI67*, and *CDK4*. The third cluster consisted of genes associated with PI3K/Akt-related signaling pathways, including *AKT1*, *PIK3CA*, *PTEN*, *MTOR*, and *EGFR*.

These bioinformatic analyses suggest that the molecular alterations observed following combined RA + Gem treatment may be associated with interconnected apoptosis-, proliferation-, and survival-related signaling networks.

These analyses were performed to identify potential functional relationships among the investigated genes and should be considered exploratory in nature. The observed interaction patterns do not establish pathway activation or mechanistic involvement in the response to RA + Gem treatment but may help identify candidate biological processes for future investigation.

#### 2.11.2. Functional GO Enrichment Analysis

Gene Ontology (GO) enrichment analysis was performed to explore potential biological functions associated with the interaction network generated from apoptosis-, proliferation-, and survival-related genes. A total of 312 significantly enriched GO terms were identified across the biological process (BP), molecular function (MF), and cellular component (CC) categories (FDR < 0.05).

**Figure 16:**
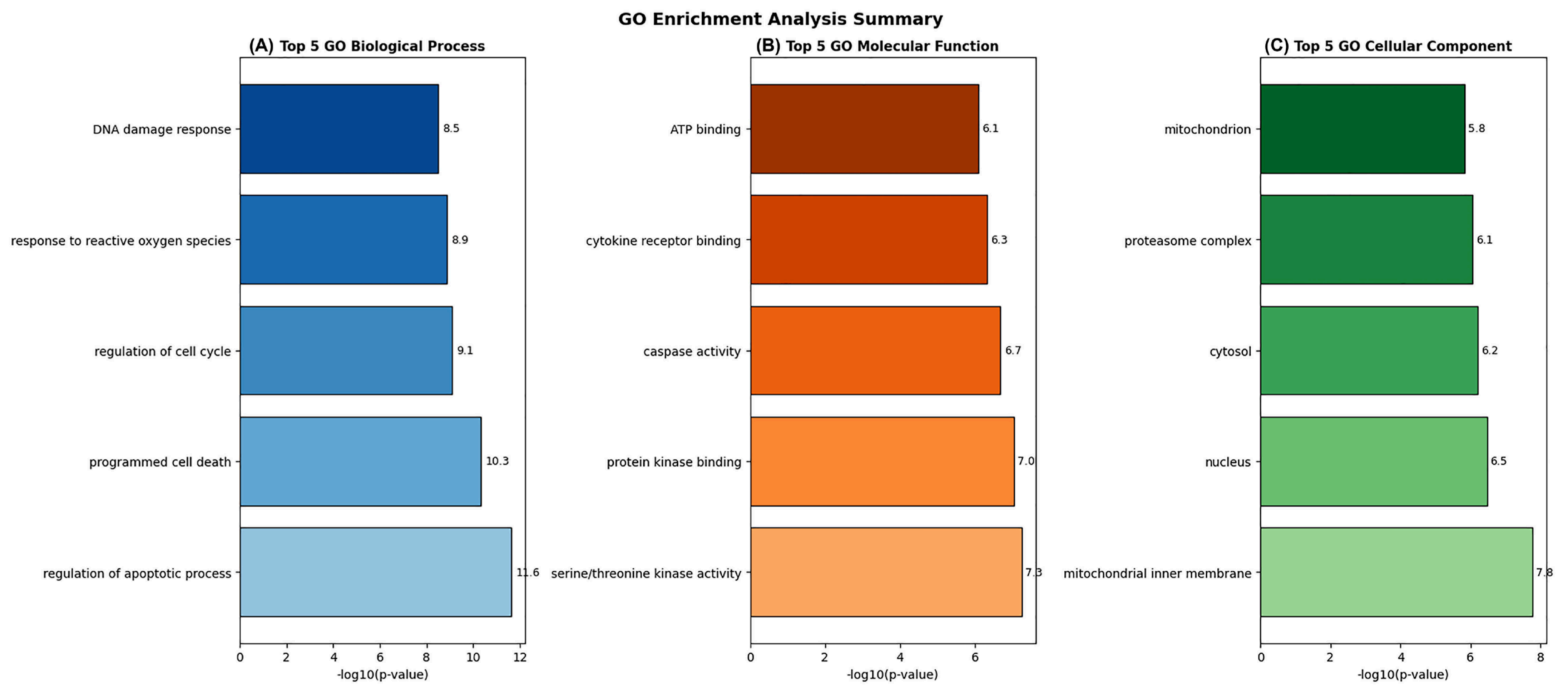
GO enrichment analysis of interaction-associated genes related to combined RA and Gem treatment in ovarian cancer cells. Bar graphs represent the top five enriched GO terms identified in the BP, MF, and CC categories. The *x*-axis represents −log10(*p*-value) enrichment scores. (**A**) Top enriched BP terms. (**B**) Top enriched MF terms. (**C**) Top enriched CC terms. The enrichment analysis is presented as an exploratory bioinformatic assessment of functional categories associated with the analyzed gene set and does not imply direct pathway activation following RA + Gem treatment.

Among the most enriched BP terms were regulation of apoptotic process (GO:0042981), programmed cell death (GO:0012501), regulation of cell cycle (GO:0051726), response to ROS (GO:0000302), and DNA damage response (GO:0006979) (Figure 16A). These enriched BP terms reflect biological processes commonly associated with the genes included in the interaction network.

In the MF category, serine/threonine kinase activity (GO:0004674), protein kinase binding (GO:0019901), caspase activity (GO:0097153), and cytokine receptor binding (GO:0005126) were among the most significantly enriched terms (Figure 16B). In the CC category, mitochondrial inner membrane (GO:0005743), nucleus (GO:0005634), cytosol (GO:0005829), and proteasome complex (GO:0000502) were identified among the enriched cellular components (Figure 16C).

Overall, these enrichment profiles provide an exploratory overview of biological processes and functional categories associated with the analyzed gene set. The results should be interpreted as hypothesis-generating and do not establish direct mechanistic involvement of these pathways in the response to combined RA + Gem treatment.

#### 2.11.3. Enriched KEGG Pathways

**Figure 17:**
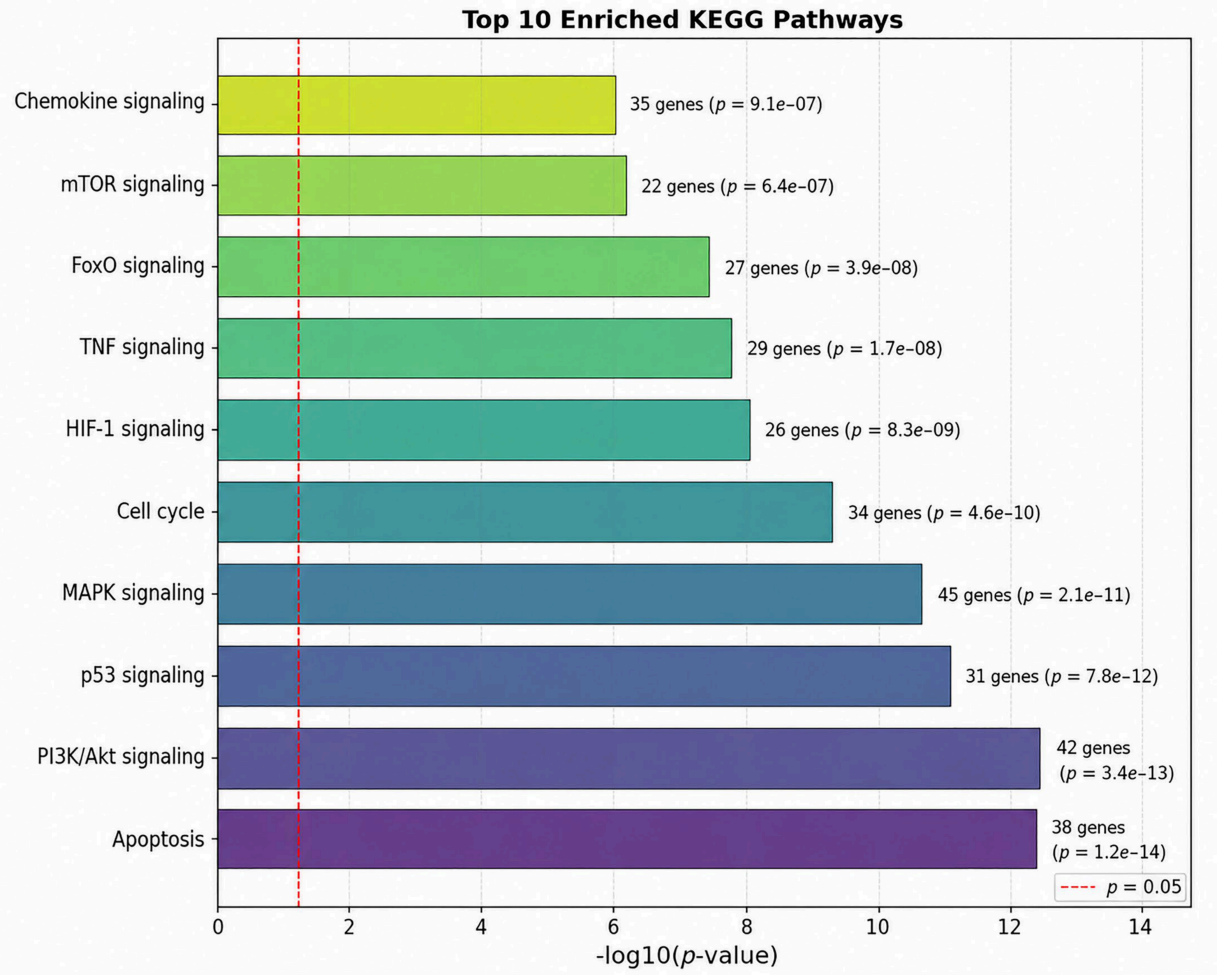
KEGG pathway enrichment analysis of interaction-associated genes related to combined RA and Gem treatment in ovarian cancer cells. The bar graph represents the top 10 significantly enriched KEGG pathways ranked according to −log10(*p*-value). Pathways associated with apoptosis, PI3K/Akt signaling, p53 signaling, MAPK signaling, and cell cycle regulation demonstrated the highest enrichment scores. The red dashed line indicates the statistical significance threshold (*p* = 0.05). The identified pathways represent bioinformatic enrichment results and are presented as exploratory, hypothesis-generating associations rather than evidence of direct pathway activation following RA + Gem treatment.

KEGG pathway enrichment analysis identified 47 significantly enriched pathways associated with the interaction network generated from apoptosis-, proliferation-, and survival-related genes (FDR < 0.05; Figure 17). The top enriched pathways included apoptosis (hsa04210; *p* = 1.2 × 10^−14^), PI3K/Akt signaling (hsa04151; *p* = 3.4 × 10^−13^), p53 signaling (hsa04115; *p* = 7.8 × 10^−12^), MAPK signaling (hsa04010; *p* = 2.1 × 10^−11^), and cell cycle regulation pathways (hsa04110; *p* = 4.6 × 10^−10^).

Additional significantly enriched pathways included HIF-1 signaling (hsa04066; *p* = 8.3 × 10^−9^), TNF signaling (hsa04668; *p* = 1.7 × 10^−8^), FoxO signaling (hsa04068; *p* = 3.9 × 10^−8^), mTOR signaling (hsa04150; *p* = 6.4 × 10^−7^), and chemokine signaling pathways (hsa04062; *p* = 9.1 × 10^−7^).

Overall, these enrichment profiles provide an exploratory overview of biological pathways associated with the analyzed gene set. The identified pathways represent bioinformatic associations and should be interpreted as hypothesis-generating rather than evidence of direct pathway activation or mechanistic involvement in the response to combined RA + Gem treatment.

## 3. Discussion

In this study, the anticancer effects of combined RA and Gem treatment were evaluated in OVCAR3 ovarian cancer cells using integrated two-dimensional (2D) and 3D experimental models together with molecular and bioinformatic analyses. The findings demonstrated that combined RA + Gem treatment produced greater reductions in cell viability and proliferative activity than either agent alone and was associated with enhanced apoptosis, cell cycle disruption, and increased intracellular ROS accumulation. NAC pretreatment partially reversed both ROS accumulation and treatment-associated cytotoxicity, supporting the involvement of oxidative stress-related mechanisms in the observed biological responses. In addition to monolayer culture findings, the inhibitory effects observed in 3D spheroid models further supported the biological activity of the combined treatment under conditions more closely reflecting tumor-like cellular organization. Molecular analyses demonstrated increased expression of proapoptotic genes (*BAX*, *CASP3*, and *CASP9*), suppression of antiapoptotic and proliferation-associated genes (*BCL2*, *MKI67*, and *CDK4*), and increased active caspase-3 immunoreactivity following combined treatment exposure. In combination, these findings suggest that RA may enhance the anticancer activity of Gem through interconnected apoptosis-, oxidative stress-, and proliferation-associated mechanisms in ovarian cancer cells while exerting comparatively less pronounced effects in non-malignant HaCaT cells.

MTT assay findings demonstrated that RA induced dose- and time-dependent reductions in cell viability in OVCAR3 cells. These observations are generally consistent with previous reports describing antiproliferative and apoptosis-associated effects of RA in breast cancer, colon cancer, and hepatocellular carcinoma models [14,22]. Yeh et al. reported apoptosis-associated effects of RA in MCF-7 breast cancer cells [23]. However, studies specifically investigating the effects of RA in ovarian cancer models remain limited, and the present findings contribute additional evidence regarding the potential biological activity of RA in this context.

Gem is a deoxycytidine analog that undergoes intracellular phosphorylation to active metabolites, which inhibit ribonucleotide reductase and interfere with DNA synthesis, resulting in replication stress, S-phase perturbation, and apoptotic cell death [4,7]. Consistent with this mechanism, cell cycle analyses in the present study demonstrated significant S-phase accumulation following Gem exposure. Importantly, combined RA + Gem treatment produced greater cytotoxic and antiproliferative effects than either agent alone. Similar observations have been reported for other natural polyphenols used in combination with conventional anticancer agents. Curcumin has been shown to enhance the efficacy of multiple chemotherapeutic drugs through chemosensitizing effects and modulation of apoptosis- and survival-related signaling pathways [24]. Likewise, resveratrol has been reported to sensitize cancer cells to anticancer drug-induced apoptosis by promoting S-phase arrest and reducing the expression of survival-associated factors, thereby enhancing treatment responsiveness [25]. Collectively, these findings support the concept that natural polyphenols can potentiate the biological activity of established chemotherapeutic agents and suggest that RA may similarly enhance the anticancer effects of Gem in ovarian cancer cells.

Flow cytometric analyses demonstrated that combined RA + Gem treatment markedly increased apoptotic cell populations in OVCAR3 cells, including both early and late apoptotic fractions. These findings were further supported by RT-qPCR and immunocytochemical analyses showing increased *BAX*/*BCL2* ratio together with elevated active caspase-3 immunoreactivity. Since mitochondrial apoptotic signaling is strongly regulated by *BAX* and *BCL2* family proteins, alterations in the balance between proapoptotic and antiapoptotic regulators may contribute to apoptosis-associated responses observed following combined treatment exposure [26]. Consistent with this interpretation, increased expression levels of *CASP3* and *CASP9* together with enhanced active caspase-3 immunoreactivity suggest the involvement of caspase-associated apoptotic signaling pathways. Similar apoptosis-associated effects of RA have also been described in other cancer models [27,28].

Cancer cells are characterized by chronically elevated basal ROS levels resulting from altered metabolism and oncogenic signaling, making them particularly susceptible to further oxidative stress [29,30]. In the present study, combined RA + Gem treatment induced substantially greater ROS accumulation in OVCAR3 cells than in non-malignant HaCaT cells. Importantly, NAC pretreatment partially reversed both intracellular ROS generation and treatment-associated cytotoxicity, supporting the contribution of oxidative stress to the biological effects of the combined treatment. Similar ROS-dependent apoptotic responses have been reported in ovarian cancer models treated with phytochemicals and targeted therapeutic agents, suggesting that modulation of redox homeostasis represents a promising therapeutic strategy [14,31,32,33,34,35]. Although ROS appears to play a major role, the incomplete rescue by NAC indicates that additional mechanisms also contribute to the anticancer activity of RA + Gem. Although EMT-related markers were not evaluated in this study, previous transcriptomic studies suggest potential interactions between ROS signaling and WNT/TGF-β-driven EMT pathways in ovarian cancer [36]. Whether these pathways contribute to the effects of RA + Gem warrants further investigation.

Bioinformatic analyses additionally provided supportive systems-level context for the experimental findings. PPI network analysis identified several highly connected genes associated with apoptosis, proliferation, and survival-related signaling pathways, including *TP53*, *AKT1*, *BCL2*, *CASP3*, *EGFR*, *MYC*, *VEGFA*, *CDK2*, *MAPK1*, and *PIK3CA*. Many of these genes have previously been implicated in ovarian cancer biology and therapeutic resistance [37,38]. KEGG enrichment analysis further demonstrated enrichment of apoptosis-, PI3K/Akt-, MAPK-, p53-, and cell cycle-associated pathways. Since these findings were generated through computational enrichment analyses, they should be interpreted as hypothesis-generating rather than direct evidence of pathway activation or inhibition. Similarly, the enrichment of mitochondria- and ROS-associated GO terms was broadly consistent with the apoptosis- and oxidative stress-related findings observed experimentally, including the NAC rescue assays [39,40].

The antiproliferative activity of combined RA + Gem treatment was further supported by reduced cell viability, suppression of MKI67 and CDK4 expression, and disruption of cell cycle progression. These findings are consistent with the observed reduction in proliferative capacity and support the concept that combined treatment inhibits both cell growth and cell cycle progression. Previous studies have similarly demonstrated that RA-based combination strategies enhance apoptosis while suppressing proliferation through modulation of stress-response and DNA damage-associated pathways [41,42,43].

The consistency of the findings obtained from both monolayer and 3D spheroid models strengthens the biological relevance of the observed anticancer effects [44]. Together, these results indicate that RA enhances Gem-induced cytotoxicity through coordinated modulation of apoptosis, oxidative stress, and proliferative signaling.

Despite several strengths, including the integration of 2D and 3D in vitro models together with apoptosis, cell cycle, oxidative stress, RT-qPCR, immunocytochemical, and bioinformatic analyses, several limitations should be acknowledged. Although NAC pretreatment experiments supported the involvement of oxidative stress in the observed cellular responses, the precise downstream molecular mechanisms remain to be fully elucidated. However, although the inclusion of 3D spheroid experiments increased the translational relevance of the study, in vitro models cannot fully reproduce the complexity of the ovarian tumor microenvironment. Furthermore, the use of a single ovarian cancer cell line (OVCAR3) limits the broader generalizability of the findings across different ovarian cancer subtypes. In addition, OVCAR3 cells harbor TP53 mutations, which may influence treatment-associated responses and limit extrapolation of the observed effects to ovarian cancers with different molecular characteristics. Additionally, the use of HaCaT keratinocytes as the non-malignant comparator cell line represents a limitation, as these cells do not originate from ovarian tissue. Therefore, future studies employing normal ovarian surface epithelial or fallopian tube epithelial cell models would provide a more tissue-specific assessment of treatment selectivity. Another limitation is that mechanistic interpretations were primarily based on gene expression, immunocytochemical, and bioinformatic analyses without extensive protein-level pathway validation. Finally, the pharmacokinetic properties, metabolic stability, and bioavailability limitations of RA remain important considerations that require further investigation in future in vivo and translational studies.

## 4. Materials and Methods

### 4.1. Cell Culture Conditions

The human ovarian adenocarcinoma cell line OVCAR3 was obtained from the American Type Culture Collection (ATCC, Manassas, VA, USA), whereas the immortalized human keratinocyte cell line HaCaT was purchased from Cell Lines Service (CLS, Eppelheim, Germany). OVCAR3 cells were cultured in RPMI-1640 medium (Gibco, Thermo Fisher Scientific, Waltham, MA, USA) supplemented with 10% fetal bovine serum (FBS; Gibco, Thermo Fisher Scientific), 1% penicillin–streptomycin solution (Gibco, Thermo Fisher Scientific), and 0.01 mg/mL insulin (Sigma-Aldrich, St. Louis, MO, USA). HaCaT cells were maintained in Dulbecco’s Modified Eagle Medium (DMEM; Gibco, Thermo Fisher Scientific) supplemented with 10% FBS and 1% penicillin–streptomycin. All cells were maintained at 37 °C in a humidified atmosphere containing 5% CO_2_ using a standard cell culture incubator (Thermo Fisher Scientific, Waltham, MA, USA). Cells were passaged using 0.25% trypsin–EDTA solution (Gibco, Thermo Fisher Scientific), and all experiments were performed using cells between passages 3 and 10.

### 4.2. Drugs and Chemicals

RA (RA; purity ≥ 98%; catalog no. R4033) and N-acetylcysteine (NAC; catalog no. A7250) were purchased from Sigma-Aldrich (St. Louis, MO, USA). Gem hydrochloride (Gem; purity ≥ 99%) was obtained from Koçak Farma (Istanbul, Türkiye). RA stock solutions were prepared in dimethyl sulfoxide (DMSO; Sigma-Aldrich) at a concentration of 100 mM, whereas Gem stock solutions were prepared in sterile phosphate-buffered saline (PBS) at a concentration of 10 mM. NAC stock solution was prepared at 100 mM in serum-free culture medium. All stock solutions were stored at −20 °C and freshly diluted in the corresponding culture medium immediately before each experiment. The final DMSO concentration in the culture medium did not exceed 0.1% in any experimental condition.

### 4.3. Cell Viability Assay and CI Analysis

Cell viability was evaluated using the 3-(4,5-dimethylthiazol-2-yl)-2,5-diphenyltetrazolium bromide (MTT) assay. OVCAR3 and HaCaT cells were seeded into 96-well plates at a density of 5 × 10^3^ cells/well and incubated for 24 h to allow cell attachment. Following incubation, cells were treated with RA (RA; 10, 20, 50, 100, 250, and 500 µM), Gem (Gem; 1, 2.5, 5, 10, 25, and 50 µM), or combined RA + Gem treatment for 24 and 48 h. Combined treatment conditions were designed according to IC_50_-based fixed-ratio concentrations determined from preliminary dose–response analyses. Control cells were incubated with culture medium containing vehicle solution (0.1% DMSO). At the end of the treatment period, 20 µL MTT solution (5 mg/mL in PBS; Sigma-Aldrich, St. Louis, MO, USA) was added to each well, followed by incubation at 37 °C for 4 h. The resulting formazan crystals were dissolved in 200 µL DMSO, and absorbance values were measured at 570 nm using a microplate reader. Cell viability was expressed as percentage relative to untreated control cells. IC_50_ values were calculated from nonlinear dose–response curves using GraphPad Prism version 9.0 (GraphPad Software, San Diego, CA, USA). Drug interaction analysis was performed according to the Chou–Talalay method using CompuSyn software (ComboSyn Inc., Paramus, NJ, USA). CI values were calculated to evaluate drug interaction profiles, where CI < 1 indicates synergy, CI = 1 indicates an additive effect, and CI > 1 indicates antagonism.

### 4.4. Flow Cytometric Analysis of Apoptosis

Apoptotic and necrotic cell populations were evaluated by flow cytometry using an Annexin V-FITC/Propidium Iodide (PI) double staining kit (BioLegend, San Diego, CA, USA). OVCAR3 and HaCaT cells were seeded into 6-well plates at a density of 2 × 10^5^ cells/well and incubated for 24 h to allow cell attachment. Cells were subsequently treated with RA at IC_50_ concentration, Gem at IC_50_ concentration, or combined RA + Gem treatment for 48 h. Following treatment, cells were harvested using trypsinization, washed twice with cold PBS, and resuspended in Annexin V binding buffer according to the manufacturer’s instructions. Subsequently, 5 µL Annexin V-FITC and 5 µL PI solution were added to each sample, followed by incubation for 15 min in the dark at room temperature. Flow cytometric analyses were performed using a BD FACSCanto II flow cytometer (BD Biosciences, San Jose, CA, USA). A minimum of 10,000 events was acquired for each sample. Data were analyzed using FlowJo software version 10.8 (BD Biosciences, Ashland, OR, USA). Cell debris and doublets were excluded based on forward- and side-scatter characteristics prior to quadrant analysis. Viable (Annexin V^−^/PI^−^), early apoptotic (Annexin V^+^/PI^−^), late apoptotic (Annexin V^+^/PI^+^), and necrotic (Annexin V^−^/PI^+^) cell populations were quantified separately.

### 4.5. Flow Cytometric Analysis of Cell Cycle Distribution

Cell cycle distribution was evaluated by flow cytometry using PI staining. Following treatment exposure, cells were harvested by trypsinization, washed twice with PBS, and fixed overnight in cold 70% ethanol at −20 °C. After fixation, cells were washed with PBS and incubated in staining solution containing 50 µg/mL PI and 100 µg/mL RNase A (Sigma-Aldrich, St. Louis, MO, USA) for 30 min at 37 °C in the dark. DNA content was subsequently analyzed using a BD FACSCanto II flow cytometer (BD Biosciences, San Jose, CA, USA). A minimum of 10,000 events was acquired for each sample. Cell populations in G0/G1, S, and G2/M phases were quantified using ModFit LT software (Verity Software House, Topsham, ME, USA). Debris and cell doublets were excluded during gating analysis.

### 4.6. Measurement of Intracellular ROS Levels

Intracellular ROS levels were determined using the fluorescent probe 2′,7′-dichlorodihydrofluorescein diacetate (DCFH-DA; Sigma-Aldrich, St. Louis, MO, USA). OVCAR3 and HaCaT cells were seeded into 6-well plates at a density of 2 × 10^5^ cells/well and incubated for 24 h to allow cell attachment. Cells were subsequently exposed to the indicated treatments for 48 h. Following treatment exposure, cells were washed with PBS and incubated with 25 µM DCFH-DA prepared in serum-free culture medium for 30 min at 37 °C in the dark. Cells were then harvested using trypsinization, washed with PBS, and analyzed using a BD FACSCanto II flow cytometer (BD Biosciences, San Jose, CA, USA). Fluorescence intensity was measured using excitation and emission wavelengths of 488 nm and 525 nm, respectively. A minimum of 10,000 events was acquired for each sample. Intracellular ROS levels were expressed as fold change relative to untreated control cells.

### 4.7. NAC Pretreatment and ROS Modulation Assay

To investigate the contribution of oxidative stress-associated mechanisms to treatment-induced cytotoxicity, NAC pretreatment experiments were performed. OVCAR3 cells were pretreated with 5 mM N-acetylcysteine (NAC) for 1 h prior to exposure to RA at IC_50_ concentration, Gem at IC_50_ concentration, or combined RA + Gem treatment. Following NAC pretreatment, cells were incubated with the indicated treatments for 48 h. At the end of the incubation period, cell viability was evaluated using the MTT assay, whereas intracellular ROS levels were measured using the DCFH-DA assay according to the procedures described above. The effects of NAC pretreatment on treatment-associated cytotoxicity and ROS accumulation were assessed by comparing viability and ROS levels between treatment-only groups and NAC-pretreated groups.

### 4.8. RNA Isolation and cDNA Synthesis

Total RNA was isolated using TRIzol reagent (Invitrogen, Thermo Fisher Scientific, Waltham, MA, USA) according to the manufacturer’s instructions. Following treatment exposure, cells were lysed in TRIzol reagent, and RNA extraction was performed by chloroform phase separation. RNA was subsequently precipitated using isopropanol, washed with 75% ethanol, and dissolved in DEPC-treated sterile water. RNA concentration and purity were evaluated using a NanoDrop 2000 spectrophotometer (Thermo Fisher Scientific, Waltham, MA, USA). Samples with A260/A280 ratios ≥ 1.8 were considered suitable for further analysis. RNA integrity was additionally verified by visualization of 28S and 18S ribosomal RNA bands following electrophoresis on 1% agarose gels. Complementary DNA (cDNA) synthesis was performed using the RevertAid First Strand cDNA Synthesis Kit (Thermo Fisher Scientific, Waltham, MA, USA) with 1 µg total RNA according to the manufacturer’s protocol.

### 4.9. Quantitative Real-Time PCR (RT-qPCR)

Gene expression analyses were performed using SYBR Green-based quantitative real-time PCR (RT-qPCR). Amplification reactions were prepared in 96-well plates using PowerUp SYBR Green Master Mix (Applied Biosystems, Thermo Fisher Scientific, Waltham, MA, USA) in a final reaction volume of 10 µL. RT-qPCR analyses were carried out using a QuantStudio Real-Time PCR System (Applied Biosystems, Thermo Fisher Scientific). Cycling conditions consisted of an initial denaturation step at 95 °C for 10 min, followed by 40 amplification cycles of 95 °C for 15 s and 60 °C for 1 min. Expression levels of apoptosis-related genes (*BAX*, *BCL2*, *CASP3*, *CASP9*, and *TP53*) and proliferation-related genes (*MKI67* and *CDK4*) were evaluated. *GAPDH* and *ACTB* were used as reference genes for normalization. Relative gene expression levels were calculated using the 2^−ΔΔCt^ method. Melting curve analysis was performed following each amplification reaction to verify amplification specificity. Primer sequences used in the study are presented in Table 1. All experiments were performed with three biological replicates and three technical replicates.

### 4.10. Immunocytochemistry

Immunocytochemical staining was performed using a DAB (3,3′-diaminobenzidine) chromogen system to evaluate active caspase-3 immunoreactivity. Cells were seeded onto sterile glass coverslips and exposed to the indicated treatments. Following treatment exposure, cells were fixed with 4% paraformaldehyde (Sigma-Aldrich, St. Louis, MO, USA) for 15 min at room temperature. To block endogenous peroxidase activity, cells were incubated with methanol containing 3% hydrogen peroxide (H_2_O_2_) for 10 min at room temperature. Cells were subsequently permeabilized with PBS containing 0.3% Triton X-100 (Sigma-Aldrich) for 10 min and blocked with 5% normal goat serum prepared in PBS for 1 h at room temperature to minimize nonspecific binding. Active caspase-3 immunostaining was performed using a rabbit anti-active caspase-3 primary antibody (1:200 dilution; catalog no. ab184787; Abcam, Cambridge, UK). Cells were incubated with the primary antibody overnight at 4 °C in a humidified chamber. Following washing steps, a biotinylated anti-rabbit IgG secondary antibody (1:500 dilution) was applied for 30 min at room temperature, followed by incubation with streptavidin–HRP complex for an additional 30 min. Color development was achieved using a DAB substrate kit (Sigma-Aldrich), and the reaction was terminated with distilled water once optimal brown color development was observed under light microscopy. Mayer’s hematoxylin (Sigma-Aldrich) was used for nuclear counterstaining. Cells were subsequently dehydrated using graded ethanol series, cleared in xylene, and mounted using Entellan mounting medium (Merck, Darmstadt, Germany). Semi-quantitative evaluation of active caspase-3 immunoreactivity was performed using the H-score method. Images were acquired under a light microscope at ×100 and ×200 magnifications, and image analyses were performed using ImageJ software (National Institutes of Health, Bethesda, MD, USA).

### 4.11. 3D Tumor Spheroid Modeling to Recapitulate In Vivo-like Drug Responses

To better recapitulate tumor architecture and microenvironmental complexity, 3D tumor spheroids were generated using the OVCAR3 ovarian cancer cell line.

#### 4.11.1. Establishment of Uniform 3D Tumor Spheroids

A 3D tumor spheroid model was established to evaluate treatment responses under conditions that mimic cell–cell interactions, nutrient gradients, and diffusion limitations characteristic of solid tumors. OVCAR3 cells were cultured in RPMI-1640 medium supplemented with 10% FBS (FBS; Gibco, Thermo Fisher Scientific, Waltham, MA, USA), 1% penicillin–streptomycin, and 0.01 mg/mL insulin. Cells in the logarithmic growth phase were seeded into ultra-low attachment round-bottom 96-well plates (Corning Inc., Corning, NY, USA) at a density of 3 × 10^3^ cells per well in a final volume of 200 µL. Plates were incubated at 37 °C in a humidified atmosphere with 5% CO_2_ to allow spontaneous aggregation and spheroid formation. Uniform and compact spheroids formed within 48–72 h and were selected for subsequent experiments. Spheroid selection was based on the presence of a regular spherical morphology, smooth and well-defined borders, and the absence of visible spontaneous fragmentation, fusion events, or satellite cell aggregates. Spheroids that did not meet these morphological criteria were excluded from subsequent analyses.

#### 4.11.2. Treatment of 3D Tumor Spheroids Under Diffusion-Limited Conditions

Following spheroid formation, culture medium was carefully replaced with fresh medium containing RA, Gem, or their combination. Drug concentrations were selected based on IC_50_ values obtained from two-dimensional monolayer dose–response experiments and were used as operational reference concentrations. To account for reduced proliferation rates and limited drug penetration within 3D structures, spheroids were exposed to treatments for 72 h under standard culture conditions. Vehicle-treated spheroids were included as controls.

#### 4.11.3. Bright-Field Imaging and Qualitative Morphological Assessment

Morphological changes in spheroids were documented using an inverted bright-field microscope (Olympus, Tokyo, Japan). Images were acquired using identical optical settings across all experimental groups to ensure comparability. Spheroid morphology was qualitatively evaluated based on structural integrity, compactness, and the presence of cellular dispersion or fragmentation.

#### 4.11.4. Quantitative Analysis of Spheroid Size

Spheroid size was quantified using ImageJ software (version 1.53; National Institutes of Health, Bethesda, MD, USA). The diameter of each spheroid was calculated as the mean of two orthogonal measurements obtained at the widest spheroid regions. Analyses were performed using spheroids derived from at least three independent biological replicates.

#### 4.11.5. Assessment of 3D Spheroid Viability Using ATP-Based Luminescence Assay

Cell viability within spheroids was determined using a luminescence-based ATP assay optimized for 3D cultures (CellTiter-Glo^®^ 3D Cell Viability Assay; Promega, Madison, WI, USA). An equal volume of reagent was added directly to each well to ensure complete spheroid lysis and ATP release. Following incubation for signal stabilization, luminescence was measured using a microplate reader (BioTek Instruments, Winooski, VT, USA). Viability values were normalized to vehicle-treated controls and expressed as percentages.

#### 4.11.6. Live/Dead Fluorescence Imaging for Spatial Assessment of Cytotoxicity

Treatment-associated cytotoxicity within spheroids was further evaluated using fluorescence-based viability staining with Calcein-AM and Ethidium homodimer-1 (Thermo Fisher Scientific, Waltham, MA, USA). Following staining, spheroids were imaged using an inverted fluorescence microscope (Olympus, Tokyo, Japan) equipped with appropriate excitation and emission filters. Images were captured at a consistent focal plane and with identical exposure settings across all experimental groups without post-acquisition image processing. Live/dead staining patterns were interpreted in conjunction with spheroid morphology to assess the spatial distribution of viable and non-viable cells within the 3D tumor structures.

### 4.12. Functional Dissection of ROS Contribution Using NAC-Based Antioxidant Rescue in 3D Tumor Spheroids

The contribution of ROS to treatment-associated cytotoxicity was functionally evaluated using an antioxidant rescue approach with NAC. 3D tumor spheroids were generated as described above and cultured until compact and morphologically uniform structures were established. Mature spheroids were pretreated with NAC (5 mM; Sigma-Aldrich, St. Louis, MO, USA) for 1 h at 37 °C in a humidified 5% CO_2_ atmosphere. This condition was selected to ensure effective intracellular ROS scavenging while minimizing nonspecific cytotoxic effects. Following pretreatment, spheroids were exposed to RA, Gem, or their combination using concentrations identical to those applied in 3D spheroid experiments. Experimental controls included untreated spheroids and spheroids treated with NAC alone. Following treatment initiation, spheroids were maintained under standard culture conditions for the indicated experimental durations prior to downstream analyses.

#### 4.12.1. Quantification of Intracellular ROS Levels Following NAC Pretreatment

Intracellular ROS levels were quantified using the fluorescent probe DCFH-DA (DCFH-DA; Sigma-Aldrich, St. Louis, MO, USA). Following treatment, spheroids were enzymatically dissociated into single-cell suspensions using Accutase^®^ (Sigma-Aldrich) to enable quantitative fluorescence analysis at the cellular level. Because ROS measurements in spheroid-derived cells were performed using a plate-reader–based fluorescence assay rather than flow cytometry, the DCFH-DA concentration was adjusted to 10 µM to ensure reliable fluorescence detection within the dynamic range of the assay. Cells were washed with PBS and incubated with DCFH-DA (10 µM) for 30 min at 37 °C under light-protected conditions. After incubation, excess probe was removed by washing with PBS. Fluorescence intensity was measured using a microplate reader (λ_excitation = 485 nm, λ_emission = 535 nm). ROS levels were expressed as fold change relative to untreated control samples, allowing comparative assessment of intracellular oxidative stress across experimental conditions.

#### 4.12.2. Evaluation of Cytotoxicity Following ROS Scavenging

The functional impact of ROS neutralization on treatment-associated cytotoxicity was evaluated using an ATP-based luminescence assay optimized for 3D spheroid cultures (CellTiter-Glo^®^ 3D Cell Viability Assay; Promega, Madison, WI, USA). Following treatment, an equal volume of reagent was added directly to each well to ensure complete spheroid disruption and ATP release. Luminescence was measured using a microplate reader, and viability values were normalized to untreated control spheroids. The extent to which NAC pretreatment attenuated ROS accumulation and partially restored spheroid viability was interpreted as a functional indicator of ROS involvement in mediating treatment-associated cytotoxic effects.

### 4.13. Bioinformatic and Network Analysis

Bioinformatic analyses were performed to investigate potential functional interactions and pathway associations among apoptosis-, proliferation-, and survival-related genes evaluated in the study. PPI network analysis was conducted using the STRING database (version 11.5; https://string-db.org/; accessed on 15 April 2026) with the organism restricted to Homo sapiens and a minimum interaction confidence score threshold of 0.700. The generated interaction network was visualized and analyzed using Cytoscape software version 3.10.0 (Cytoscape Consortium, San Diego, CA, USA). Functional cluster analysis within the PPI network was performed using the Molecular Complex Detection (MCODE) plugin in Cytoscape. GO enrichment analysis, including BP, MF, and CC categories, as well as Kyoto Encyclopedia of Genes and Genomes (KEGG) pathway enrichment analysis, were performed using the Enrichr platform (https://maayanlab.cloud/Enrichr/; accessed on 15 April 2026). Enrichment significance was evaluated using false discovery rate (FDR)-adjusted *p*-values, and terms/pathways with FDR < 0.05 were considered statistically significant.

#### 4.13.1. PPI Network Analysis

An integrated bioinformatic workflow was applied to predict potential molecular targets associated with RA, Gem, and ovarian cancer. Putative compound-associated targets of RA and Gem were identified using the SwissTargetPrediction database (http://www.swisstargetprediction.ch; accessed on 15 April 2026) and the PharmMapper platform (http://www.lilab-ecust.cn/pharmmapper/; accessed on 15 April 2026). Ovarian cancer-associated genes were retrieved from the GeneCards database (https://www.genecards.org/; accessed on 15 April 2026) and the Online Mendelian Inheritance in Man (OMIM) database (https://www.omim.org/; accessed on 15 April 2026). Shared candidate genes identified from compound-associated and disease-associated datasets were subsequently used for PPI network analysis. The PPI network was constructed using the STRING database (version 11.5; https://string-db.org/; accessed on 15 April 2026) with the organism restricted to Homo sapiens and a minimum interaction confidence score threshold of 0.700 (high confidence). The resulting interaction network was visualized and analyzed using Cytoscape software version 3.10.0 (Cytoscape Consortium, San Diego, CA, USA).

#### 4.13.2. GO Enrichment Analysis

GO enrichment analysis was performed to investigate potential biological functions associated with the interaction network genes identified in the study. GO analyses were conducted using the clusterProfiler R package and categorized into BP, MF, and CC ontologies. Significantly enriched GO terms were identified based on FDR-adjusted *p*-values < 0.05. Enrichment results were ranked according to statistical significance and gene enrichment profiles. The top enriched GO categories associated with apoptosis, oxidative stress response, cell cycle regulation, kinase activity, and mitochondrial CCs were selected for visualization and interpretation. GO enrichment results were visualized using bar plots generated in R software (version 4.5.1; R Foundation for Statistical Computing, Vienna, Austria), and enrichment scores were expressed as −log10(*p*-value).

#### 4.13.3. KEGG Pathway Enrichment Analysis

KEGG pathway enrichment analysis was performed to explore potential biological pathways associated with the interaction network genes identified in the study. Analyses were conducted using the enrichKEGG (version 4.5.1; R Foundation for Statistical Computing, Vienna, Austria) function implemented in the clusterProfiler R package with the organism code specified as “hsa” (*Homo sapiens*). Significantly enriched pathways were identified based on FDR-adjusted *p*-values < 0.05 and enrichment score (GeneRatio) ≥ 0.1. Enriched pathways included apoptosis, PI3K/Akt signaling, p53 signaling, MAPK signaling, and cell cycle-associated pathways. Pathway enrichment results were visualized using bar plots and dot plots and ranked according to gene count, enrichment score, and statistical significance. In addition, the Pathview package (version 1.38.0) was used to visualize pathway-associated genes within KEGG pathway diagrams.

### 4.14. Statistical Analysis

All experiments were performed using at least three independent biological replicates (*n* ≥ 3). Each biological replicate represented an independently performed experiment using separately cultured cells. Quantitative data are presented as mean ± standard deviation (SD). Statistical analyses were performed using GraphPad Prism version 9.0 (GraphPad Software, San Diego, CA, USA). Comparisons among multiple experimental groups were performed using one-way analysis of variance (ANOVA) followed by Tukey’s multiple comparison post hoc test. Normality assumptions were evaluated using the Shapiro–Wilk test prior to parametric analyses. A *p*-value < 0.05 was considered statistically significant. For RT-qPCR experiments, each biological replicate was analyzed with three technical replicates. Flow cytometry data were analyzed from a minimum of 10,000 acquired events per sample. CI analyses based on the Chou–Talalay method were interpreted descriptively according to standard CI thresholds rather than inferential statistical testing. For immunocytochemical H-score evaluation, scoring consistency between observers was assessed using ICC analysis.

## 5. Conclusions

The present study demonstrated that combined RA and Gem treatment produced enhanced antiproliferative and apoptosis-associated effects in OVCAR3 ovarian cancer cells compared with single-agent exposure conditions. Integrated findings obtained from 2D and 3D experimental models revealed that the combined treatment was associated with increased intracellular ROS accumulation, disruption of cell cycle progression, suppression of proliferation-associated gene expression, and enhanced apoptotic responses. Partial reversal of cytotoxicity and ROS accumulation following NAC pretreatment further supported the involvement of oxidative stress-related mechanisms in the observed cellular responses.

Molecular and immunocytochemical analyses demonstrated increased expression of proapoptotic genes (*BAX*, *CASP3*, and *CASP9*), suppression of proliferation-associated markers (*MKI67* and *CDK4*), and elevated active caspase-3 immunoreactivity following combined treatment exposure. In addition, bioinformatic analyses identified enrichment of apoptosis-, oxidative stress-, PI3K/Akt-, and cell cycle-associated signaling pathways, providing supportive systems-level context for the experimental findings. The inhibitory effects observed in 3D spheroid models further strengthened the biological relevance of the combined treatment under conditions partially reflecting tumor-like cellular organization.

In summary, these findings suggest that RA may enhance the anticancer activity of Gem through interconnected oxidative stress-, apoptosis-, and proliferation-associated mechanisms in ovarian cancer cells. However, since the present findings are limited to in vitro experimental systems, additional in vivo, pharmacokinetic, and protein-level mechanistic studies are required to further evaluate the therapeutic potential and translational applicability of this combinational approach in ovarian cancer.

## Figures and Tables

**Table 1:** Primer sequences.

Gene	Forward Primer (5′→3′)	Reverse Primer (5′→3′)
*CASP3*	GGAAGCGAATCAATGGACTCTGG	GCATCGACATCTGTACCAGACC
*CASP9*	GTTTGAGGACCTTCGACCAGCT	CAACGTACCAGGAGCCACTCTT
*BAX*	TCAGGATGCGTCCACCAAGAAG	TGTGTCCACGGCGGCAATCATC
*BCL2*	ATCGCCCTGTGGATGACTGAGT	GCCAGGAGAAATCAAACAGAGGC
*TP53*	CCTCAGCATCTTATCCGAGTGG	TGGATGGTGGTACAGTCAGAGC
*MKI67*	GAAAGAGTGGCAACCTGCCTTC	GCACCAAGTTTTACTACATCTGCC
*CDK4*	CCATCAGCACAGTTCGTGAGGT	TCAGTTCGGGATGTGGCACAGA
*ACTB* (*β-actin*)	CATTGCTGACAGGATGCAGAAGG	TGCTGGAAGGTGGACAGTGAGG
*GAPDH*	GGAGCGAGATCCCTCCAAAAT	GGCTGTTGTCATACTTCTCATGG

## Data Availability

The original contributions presented in this study are included in the article. Further inquiries can be directed at the corresponding author.

## Abbreviations

ACTB	Actin Beta
ANOVA	Analysis of Variance
ATP	Adenosine Triphosphate
BAX	BCL2 Associated X Protein
BCL2	B-Cell Lymphoma 2
BP	Biological Process
CASP3	Caspase 3
CASP9	Caspase 9
CC	Cellular Component
CDK4	Cyclin-Dependent Kinase 4
CI	Combination Index
DAB	3,3′-Diaminobenzidine
DCFH-DA	2′,7′-Dichlorodihydrofluorescein Diacetate
DMEM	Dulbecco’s Modified Eagle Medium
DMSO	Dimethyl Sulfoxide
DRI	Drug Reduction Index
EMT	Epithelial–Mesenchymal Transition
FBS	Fetal Bovine Serum
FDR	False Discovery Rate
Gem	Gemcitabine
GO	Gene Ontology
ICC	Intraclass Correlation Coefficient
IC_50_	Half-Maximal Inhibitory Concentration
KEGG	Kyoto Encyclopedia of Genes and Genomes
MF	Molecular Function
MKI67	Marker of Proliferation Ki-67
MCODE	Molecular Complex Detection
MTT	3-(4,5-Dimethylthiazol-2-yl)-2,5-Diphenyltetrazolium Bromide
NAC	N-Acetyl-L-Cysteine
PBS	Phosphate-Buffered Saline
PI	Propidium Iodide
PPI	Protein–Protein Interaction
qRT-PCR/RT-qPCR	Quantitative Real-Time Polymerase Chain Reaction
RA	Rosmarinic Acid
RNA	Ribonucleic Acid
ROS	Reactive Oxygen Species
SD	Standard Deviation
STRING	Search Tool for the Retrieval of Interacting Genes/Proteins
TP53	Tumor Protein p53
2D	Two-Dimensional
3D	Three-Dimensional
